# A new theory for X-ray diffraction

**DOI:** 10.1107/S205327331400117X

**Published:** 2014-03-27

**Authors:** Paul F. Fewster

**Affiliations:** a PANalytical Research Centre, Sussex Innovation Centre, Falmer, Brighton, East Sussex BN1 9SB, UK

**Keywords:** diffraction theory, powder diffraction, small crystals

## Abstract

This article proposes a new theory of X-ray scattering that has particular relevance to powder diffraction. The underlying concept of this theory is that the scattering from a crystal or crystallite is distributed throughout space: this leads to the effect that enhanced scatter can be observed at the ‘Bragg position’ even if the ‘Bragg condition’ is not satisfied. The scatter from a single crystal or crystallite, in any fixed orientation, has the fascinating property of contributing simultaneously to many ‘Bragg positions’. It also explains why diffraction peaks are obtained from samples with very few crystallites, which cannot be explained with the conventional theory. The intensity ratios for an Si powder sample are predicted with greater accuracy and the temperature factors are more realistic. Another consequence is that this new theory predicts a reliability in the intensity measurements which agrees much more closely with experimental observations compared to conventional theory that is based on ‘Bragg-type’ scatter. The role of dynamical effects (extinction *etc.*) is discussed and how they are suppressed with diffuse scattering. An alternative explanation for the Lorentz factor is presented that is more general and based on the capture volume in diffraction space. This theory, when applied to the scattering from powders, will evaluate the full scattering profile, including peak widths and the ‘background’. The theory should provide an increased understanding of the reliability of powder diffraction measurements, and may also have wider implications for the analysis of powder diffraction data, by increasing the accuracy of intensities predicted from structural models.

## Abstract 2

By considering the scattering distributed throughout space, there is an intensity enhancement at the Bragg angle even when the Bragg condition is not satisfied. This leads to an alternative explanation for the diffraction from powders and small crystals.

## 1. Introduction   

The concept of Bragg’s law assumes that the scattering is concentrated at discrete points and that away from these positions the mutual interference gives no significant scattering (Bragg, 1925 ▶). An alternative viewpoint is presented here, where the whole of diffraction space is occupied by scattering from many crystal planes, which when combined contribute to the peaks observed. This effect is most obvious in X-ray powder diffraction and this is therefore the main focus of this article.

X-ray powder diffraction was pioneered by Debye & Scherrer (1916 ▶) and is now a well established technique that has been used successfully for nearly 100 years. This is a very important technique for the identification of material phases and their quantitative proportion, microstructure evaluation and molecular structure determination. A powder is in general an accumulation of small crystallites with dimensions ∼10 µm or less. Most materials have some identifiable atomic periodicity and therefore create an X-ray diffraction pattern. This gives X-ray powder diffraction an important role in many industries from building materials, pharmaceuticals, mining, forensic analysis *etc.*, to scientific studies on the evaluation of the microstructure and the determination of the stereo-chemistry of molecular structures. These analyses give information on the strength of materials, liability to cracking in structures, identification of polymorphs in drug design, identification of phases and their proportions in paints and cement *etc*. Its impact worldwide has been enormous and many important processes depend on X-ray powder diffraction. However, the ‘standard explanation’ of the diffraction process raises some concerns: for example, the low probability of Bragg scattering (Fewster, 2000 ▶), the high variability in peak shapes depending on experimental procedure (Fewster & Andrew, 1999 ▶) and the poor reliability of intensity measurement based on crystal statistics (Smith, 1999 ▶). Despite these concerns, the method seems to work. It is the position, width and intensity of the diffraction peaks that yield the information for the analyses mentioned above.

This article will give a brief outline of the conventional theory of X-ray powder diffraction and its shortcomings, including the theoretical estimates of crystal statistics and estimates of temperature factors, followed by an alternative theory that addresses these weaknesses. Attention will be drawn to the relevance of dynamical and kinematical scattering and the origin of the intensity, the improvement in the intensity estimates when compared with measured values and the complex nature of the intensity distribution. The whole process, based on this alternative theory, is much more subtle and fascinating than can be explained by the simple application of Bragg’s law. This improved understanding has led to, and may in the future lead to further, new diffractometer designs, more robust analyses of the data and a firmer basis for establishing the reliability of the method.

## 2. The conventional theory   

The expression for the diffracted intensity from powders has been presented by several authors and has the form given by
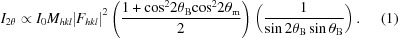
This expression can be derived based on a flat-plate detector set normal to the incident beam after passing through a small cluster of crystallites, *e.g.* Zachariasen (1945 ▶), and also is given by James (1962 ▶) for the basic single-crystal diffraction process, and by Brown (1955 ▶) for a cluster of crystallites as in the case discussed. The constant of proportionality includes absorption, wavelength effects, specimen-to-detector radius, classical electron radius *etc.*, which are all constant in a typical X-ray diffraction experiment. In the above equation, *I*_0_ is the incident-beam intensity, θ_B_ and θ_m_ are the Bragg angles for the sample and the monochromator crystal, if one exists, respectively, and *M_hkl_* is the multiplicity (the number of times a similar reflection occurs by symmetry). *F_hkl_* is the structure factor and is the coherent sum of the scattering *f_r_* from all the atoms, located at fractional coordinates *x_i_*, *y_i_*, *z_i_* in the unit cell repeat through the structure; it is given by
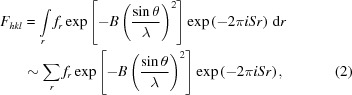
where *r* = *hx_j_* + *ky_j_* + *lz_j_*, |*S*| = 2 sin θ/λ, *B* is the Debye–Waller factor (Debye, 1913 ▶; Waller, 1923 ▶) and λ is the wavelength.

The first bracketed term in equation (1) takes account of the reduction in the intensity of one of the polarization components (π polarization) in the plane of the scattering, and the second term is a combination of the Lorentz factor and a geometric factor. The Lorentz factor is expressed as the time for the reflection to stay in the ‘Bragg condition’, and one of the first texts to discuss this was by Debye (1913 ▶) and a detailed derivation was given in Buerger (1940 ▶). Based on this description it is not applicable to any arbitrary position in diffraction space, but only at the ‘Bragg condition’. This term can be expressed as 1/sin 2θ_B_. The geometric term, 1/sin θ_B_, is related to the range of crystal plane tilts that will give rise to measured scattering through a fixed detector aperture (this can be visualized by recognizing that a large crystal plane tilt is required to rotate the scattered beam across the detector aperture at low scattering angles).

The intensity formula, equation (1), contains no information about the peak widths and is considered as the intensity associated with the ‘Bragg condition’. The peak shapes are superimposed on this ‘stick pattern’ to smear the intensity and form the profiles for comparison with the measured profiles. The peak shapes are often considered as a fitted parameter containing some mixture based on Lorentzian and Gaussian forms, *e.g.* Pearson VII, pseudo-Voigt (see, for example, Young, 1993 ▶). The peak widths are usually fitted to a quadratic tangent function (Caglioti *et al.*, 1958 ▶) which represents the varying instrument function over the experimental scan angle. The Caglioti function was derived for neutron diffractometry, assuming Gaussian profiles, and does not include the full instrument response. These functions do not account for the intensities between the peaks, unless more parameters are included that can be attributed to the sample.

The same formula, equation (1), is used for Bragg–Brentano focusing geometry as well as the Debye–Scherrer geometry; however, this does use an assumption that alters the relative intensities for the two geometries, but it is a small effect. The validity of this assumption is discussed later. The whole expression relies on a considerable degree of averaging and assumes that the diffraction profile comes purely from the crystallites that are in the Bragg condition (Bragg, 1913 ▶, 1925 ▶).

## 3. The problem with conventional theory   

The conventional theory of X-ray powder diffraction is based on the scattering at the Bragg condition for each crystalline plane, and assumes that there are sufficient crystallites in the correct orientation to create the pattern observed. At first sight this seems reasonable, since generally there could be millions of crystallites illuminated at any one time using standard instrumentation. This assumption will be considered in more detail. The geometry of the instrument along with the crystallite orientation distribution will give an estimate of the intensities for the scattering peaks.

The conventional understanding of powder diffraction will be considered in terms of:

(i) the likelihood of scattering at the Bragg condition,

(ii) the influence of sample movement,

(iii) the influence of dynamical and kinematical scattering,

(iv) the estimation of the temperature factors,

(v) an experiment illustrating the serious deficiency of conventional theory.

These aspects will be considered briefly at this stage with an emphasis on some experimental pointers and then be considered in detail later in the article.

### 3.1. The likelihood of scattering in the Bragg condition   

**Figure 1:**
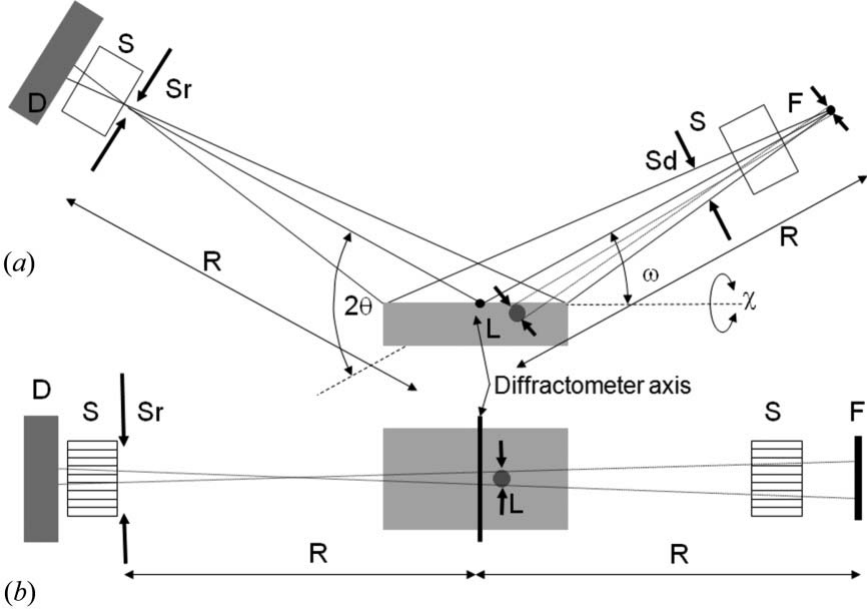
(*a*) The Bragg–Brentano geometry with a sample including crystallites of diameter L, which experience an angular spread defined by L and the focus dimension F. (*b*) is the projection normal to (*a*). R is the radius, S are the Soller slits that control the axial divergence, Sr is the receiving slit, D the detector and Sd the divergence slit.

If we consider two well characterized sample types, Si and LaB_6_ used in this study, it is possible to estimate the chance of scattering in the Bragg condition with the conventional Bragg–Brentano diffractometer (Bragg, 1921 ▶; Brentano, 1946 ▶). The geometry of the Bragg–Brentano diffractometer is given in Fig. 1 ▶(*a*). With this para-focusing geometry the sample area illuminated can be very large. A typical instrument source-to-sample distance is 240 mm with a beam width of 10 mm. Within the plane of the diffractometer, the X-ray source focus dimension could be typically 40 µm, with the divergence slit set at 0.25° and the acceptance slit at 0.25°. Alternatively, the area illuminated could be fixed at 10 × 10 mm. An approximate calculation is performed for both configurations.

The Si sample is composed of 10 µm perfect crystalline spheres and most of the scattering comes from the top 30 µm. The absorption length for Si is ∼63 µm, then for a perfectly packed sample the total number of crystallites illuminated by the incident beam would be ∼2 406 000 at 15° 2θ, ∼1 212 000 at 30° 2θ, falling to ∼618 000 at 60° 2θ. For a fixed incident-beam area the number of illuminated crystallites is 3 000 000 at all angles. For LaB_6_ the absorption length is ∼1 µm and the crystallite dimension is 3 µm. Therefore the numbers of crystallites illuminated are ∼8 880 000 at 15° 2θ, ∼4 470 000 at 30° 2θ, falling to ∼2 310 000 at 60° 2θ, assuming that the penetration is not greater than 3 µm. For a fixed area of 10 × 10 mm, the number of crystallites illuminated is 11 100 000. In practice, though, these are overestimates, but give a working value to illustrate the problem with the conventional theory. These values also assume that there is no loss of intensity through scattering; these are extinction effects incorporated into dynamical theory and could reduce the absorption depth further, and this would vary with reflecting power.

If the scattering from an individual crystallite is considered, then the divergence it experiences is calculated from the geometry of Fig. 1 ▶(*a*), for the scattering normal to the diffractometer axis. For a 10 µm crystallite, 40 µm focus and a radius of 240 mm the accepted divergence is 0.017° and for a 3 µm crystallite this is 0.01°. In the plane normal to Fig. 1 ▶(*a*), given in Fig. 1 ▶(*b*), there will also be some axial divergence that is restricted by Soller slits; for this example a typical divergence control could be based on 0.04 radian Soller slits. The axial divergence parallel to the diffractometer axis is defined by the Soller slit (or the detector dimension) geometry in Fig. 1 ▶(*b*). The probability of this geometry capturing scatter that satisfies the Bragg condition, for a specific reflection from one crystallite, is given by the product of these orthogonal accepted divergences by the crystallite compared to 4π. This can be visualized as the small acceptance region on the surface of a sphere representing the distribution of orientations compared to that of the whole sphere surface. Therefore for the geometry in Fig. 1 ▶ the number of crystallites, *X*, that are involved in contributing to the intensity through Bragg scattering for a sample with *N* (= 2 406 000, 1 212 000 and 618 000 at 15°, 30° and 60° 2θ) 10 µm Si crystallites is approximately
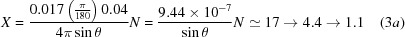
(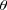 = 15° 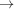 30° 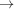 60°). And for 3 µm closely packed LaB_6_ crystallites 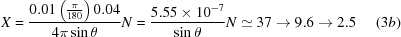
(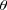 = 15° 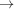 30° 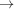 60°). Therefore with this large number of crystallites, on average there will be very few that satisfy the Bragg condition for any specific reflection. The 1/sin θ equates to the 1/sin θ_B_ term in equation (1). The counting statistics would be very poor, and going to high angular resolution instruments this would create very unreliable data. Smith (1999 ▶) estimated the unreliability in the data and concluded that any accurate analysis of minor phases in a mixture would be impossible, but was assuming thousands of millions of crystallites were contributing. However, the intensities are reproducible, which is difficult to reconcile with the assertion that the scattering only comes from the ‘Bragg condition’. To account for this apparent reliability, Alexander *et al.* (1948 ▶) had to assume that the crystallites were quite imperfect and had to diffract over considerably wider angles than their expected perfect width, whereas de Wolf (1958 ▶) assumed that the instrumental broadening was a significant contributor.

Any single crystallite will have many hundreds of possible reflections. Take for example the standard reference material LaB_6_, a cubic structure, which has 690 possible reflections accessible with Cu *K*α_1_ radiation. The number of observable diffraction peaks is 25; due to symmetry, however, it must be assumed that a significant number of reflections contribute to the 25 2θ positions to obtain reproducible intensity ratios. This still puts great demands on the range of crystallite orientations, despite having a small lattice parameter and therefore few possible reflections. For more complex structures, with lower symmetry and many thousands of reflections, the number of crystallites required to satisfy the Bragg condition for all the possible reflections, based on the conventional theory, will become very large or the intensities would be very unreliable.

### 3.2. The effect of sample movement   

Introducing sample rotation within the beam certainly improves the reliability in the intensities; however, a stationary sample can still produce all the reflections in the diffraction pattern and give very reproducible intensity ratios. Sample rotation in the Bragg–Brentano geometry increases the angular spread impinging on the sample, and the probability of satisfying the Bragg condition, by a very small amount, mainly because only those crystallites with the appropriate plane closely parallel to the surface will produce Bragg diffraction.

### 3.3. The influence of dynamical and kinematical scattering   

Depending on the material, there are significant differences in the calculated intensities when using kinematical and dynamical theories. For example, LaB_6_, *hkl* = 003, and Si, *hkl* = 004, both with crystallite dimensions of 3 µm will introduce a peak intensity difference of ∼20% (the kinematical scattering theory overestimates), which reduces with decreasing crystallite dimensions. The stronger reflections of LaB_6_, *e.g. hkl* = 110, have considerable dynamical effects and the calculated kinematical peak intensity is ×60, for this 3 µm dimension, compared with that calculated from dynamical theory. The scattering from the Bragg condition must therefore be included or reasons given as to why these dynamical effects are suppressed. However, the intensities derived using equation (1) that is based on kinematical theory fit reasonably well, and so an explanation for the suppression of dynamical effects is required. Darwin (1922 ▶) considered various crystal imperfections to suppress the dynamical effects: the most likely description was termed the mosaic crystal, sometimes termed ‘ideally imperfect’, which effectively consists of small blocks of perfect crystal that have slightly different orientations with respect to each other (Zachariasen, 1945 ▶). The size of blocks has to be small enough to suppress dynamical effects. As shown in the example above, the blocks need to be very small, probably sub-micron, for kinematical theory to be valid.

The conventional theory does not in general include refractive-index effects, although this has been discussed by Wilson (1940 ▶) for powders, whereas dynamical theory includes them naturally. However, the neglect of the refractive index makes for a very small displacement of the peaks, ∼0.005° for a flat plate, which is close to the maximum value at normal incident angles and can be 0.0° for entry and exit through surfaces normal to the scattering planes. It is debatable therefore whether the refractive index should be included to estimate the peak positions in powder diffraction. This maximum displacement of the peaks is ∼10% of the intrinsic scattering width of the profiles in a typical powder diffraction experiment. Hart *et al.* (1988 ▶) have discussed the ratio of transmission to reflection geometry based on scattering in the symmetrical ‘Bragg condition’ and estimated that it is dominated by transmission geometry. This would suggest that the refraction effect would be small and cannot account for the measured difference in lattice parameters between polycrystalline Si and bulk Si (Hubbard *et al.*, 1975 ▶). Another important conclusion of the work of Hart *et al.* is that the refractive index is negligible at large extinction distances: the extinction distance is smallest for intense Bragg reflections that have the largest dynamical effects.

### 3.4. Estimation of the temperature factors in Si   

**Figure 2:**
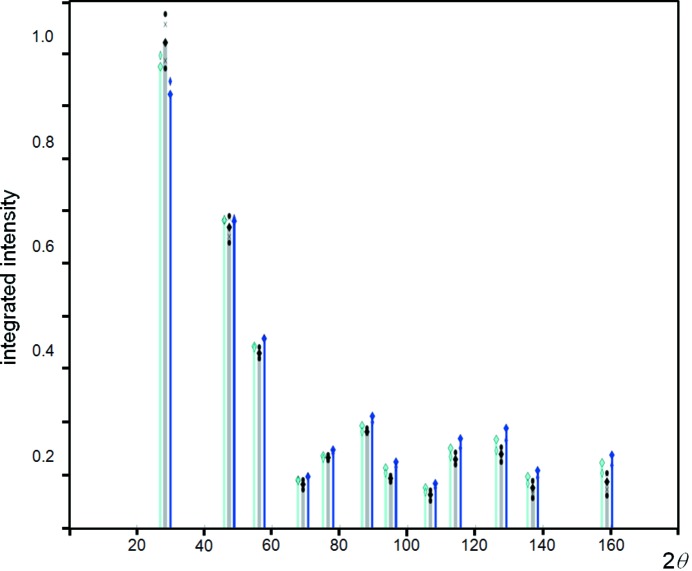
The integrated intensity for the measured reflections (grey central bars) displayed as a bar graph for an Si sample (Si crystallites immersed in a resin that occupy 50% of the volume), compared with the conventional theory: blue right-hand bars, include dynamical effects, cyan left-hand bars based purely on kinematical theory (large diamonds for *B* = 0.02 Å^−2^, small diamonds for *B* = 0.046 Å^−2^). The experimental data were collected on an instrument with *R* = 320 mm, divergence slit = 0.125°, a PIXcel detector with 255 strips of 0.055 mm and Soller slits of 0.04 radian and Cu *K*α. The black diamond is the mean of six measurements on different samples, the ‘x’ marks are the standard deviation points and ‘o’ symbols the range.

Carefully collected experimental data sets for Si powder, using Cu *K*α_1_ in para-focusing geometry, exhibit highly reproducible intensity values and ratios; however, on applying the intensity formula in equation (1), the relative intensities appear slightly overestimated with increasing 2θ angle (Fig. 2 ▶). If the dynamical effects discussed in the previous section are included then the agreement in the intensities is considerably worse. This difference can be resolved by allowing the Debye–Waller factor to increase, *e.g.* to *B* ∼ 0.06 nm^2^, if the scattering follows kinematical theory, and *B* ∼ 0.08 nm^2^ if it follows dynamical theory. This *B* factor is difficult to reconcile with the expected value of ∼0.02 nm^2^ (Reid & Pirie, 1980 ▶). The experimentally measured parameter on bulk Si of *B* ∼0.046 nm^2^, by Aldred & Hart (1973 ▶), represents the maximum value, because their experimental conditions were highly biased towards the core value. Reid & Pirie (1980 ▶) have critically reviewed the literature as well as having calculated this value for Si by numerous approaches, and concluded that *B* ∼0.02 nm^2^ is the most likely value. The structure of Si is known and therefore there is little room for manoeuvre. The *B* factor is not the complete description of thermal effects on the intensities, because this relates to the averaging of uncorrelated atomic vibrations and assumes the vibrations are isotropic. The discussion on the Debye–Waller factor is given in the Appendix[App appa].

### 3.5. An experiment illustrating a serious deficiency of conventional theory   

**Figure 3:**
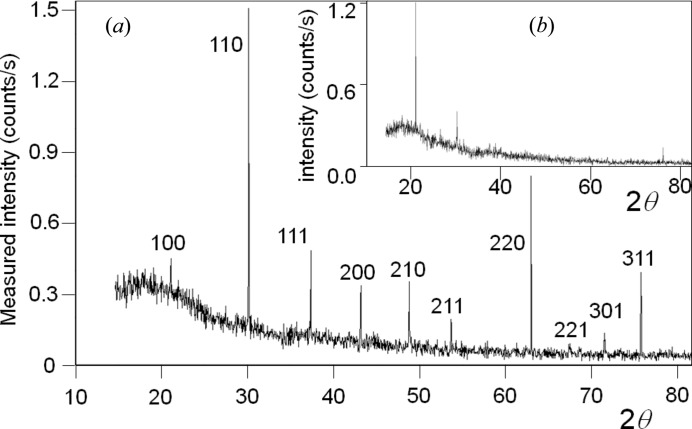
The scattering pattern from ∼120 crystallites or if perfectly packed 300 crystallites (3.5 µm beam width × 1 mm sample size and a single layer of crystallites) of LaB_6_ with crystallite sizes varying from 2 to 5 µm with the full range of reflections up to 80° 2θ (*a*). (*b*) gives the profile with ∼30 crystallites or if perfectly packed 75 crystallites (3.5 µm beam width × 0.25 mm sample size), where not all the reflections are clearly resolved as in the larger sample size. The data were collected with a 0.01° divergent Cu *K*α_1_ beam from a 1.8 kW X-ray laboratory source and a stationary sample in 35 min.

Suppose a sample with very few crystallites is studied: it is expected that the chances of observing any scattering is very unlikely, based on crystallites satisfying the ‘Bragg condition’. However, as can be seen in Fig. 3 ▶(*a*), for <300 crystallites and no sample movement, there is a very clear diffraction pattern from LaB_6_, with all the possible reflections being observed in the angular range of the experiment. With so few crystallites, a single crystal plane (*hkl*) will have on average an angular separation between different crystallites of ∼11°. This is clearly outside the range of probability for capture. This experiment used a pure Cu *K*α_1_ incident beam (3.5 µm FWHM × 1000 µm) with an angular divergence of 0.01° and a Soller slit acceptance of 2.3° (0.04 radian) (Fewster & Trout, 2013 ▶). The sample consisted of a single layer of crystallites; if the crystallites covered the adhesive mounting tape used, with no gaps, then the crystallite number would be ∼300. From X-ray absorption measurements this is unlikely and ∼40% coverage is typical, giving a crystallite number ∼120. A more extreme example is given for a sample of ∼30 crystallites or for a perfectly packed sample ∼75 crystallites (Fig. 3 ▶ *b*). The intrinsic scattering half-height width for a 3 µm crystallite is of the order of 0.002°, which is small compared with the capture volume and so has little influence on the capture likelihood.

This experiment will also give an estimate of the intensity impinging on a single crystallite. The direct beam has a flux of 10 000 photons per second on a 10 µm circular cross section, within a divergence of 0.017°. If it is assumed that the half-height width of a scattering peak is 0.002°, then each crystallite in the Bragg condition should scatter ∼1176 × *R* photons [= 10 000 × (0.002/0.017) × *R*], where *R* is the reflectivity. Suppose the reflection being studied is the 220 from Si, then from dynamical theory *R* ∼ 96% for 10 µm assuming a simplified model (for 3 µm crystallites *R* falls to 73%), giving an expected intensity of 1130 photons for each Bragg condition satisfied. This expected intensity is ∼100× greater than the total intensity gathered for this reflection from 2200^1^ crystallites, assuming a 40% coverage.

In summary, the intensity is captured for all peaks and contradicts the notion that there must be a statistically large enough number of crystallites, for a sufficient number of reflections to be in the ‘Bragg condition’. Furthermore if the observed reflections satisfied the ‘Bragg condition’, then they would be expected to be >100× more intense than they actually are. Both these observations call into question the idea that the reflections must satisfy the ‘Bragg condition’, *i.e.* arise purely from the condition when crystallites are orientated exactly for Bragg’s law to be obeyed.

## 4. An alternative explanation   

If the whole diffraction process is considered as an interference problem then the contributions are not confined to the Bragg condition. To describe the concept, the scattering is treated kinematically initially, *i.e.* there is no inclusion of dynamical effects, for example extinction (incident energy loss through scattering) and refraction effects (the refractive index of typical materials with X-rays is ∼0.9999).

The profile calculations based on dynamical and kinematical theories are coincident remote from the Bragg condition, when the refraction correction is ignored. The kinematical profile at the Bragg condition is slightly asymmetric for strong reflections when absorption is considered; however, when this is ignored, the profile matches that of a sine cardinal (sinc function). The sinc function is a very convenient way of representing the scattering profile by just considering the path differences of possible scattered beams; therefore sinc functions are used to illustrate the basic concepts in this article, although as will be shown later dynamical theory is included.

## 5. The derivation of the amplitudes   

**Figure 4:**
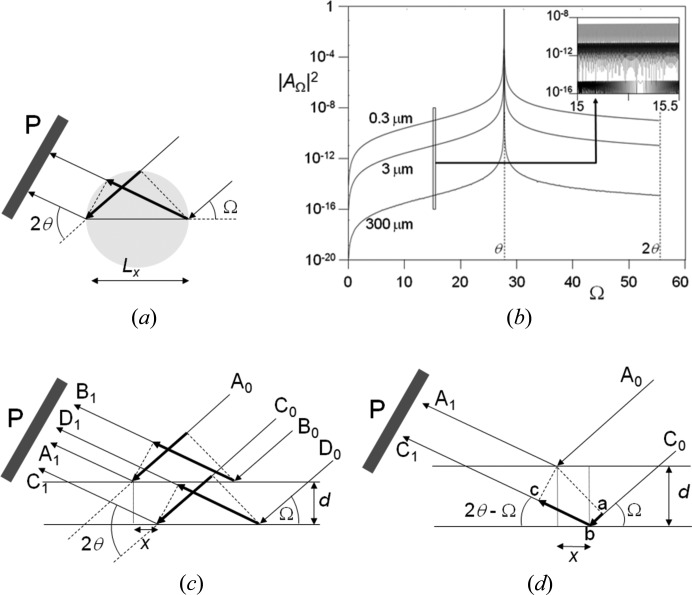
(*a*) The different path lengths for a photon from a single crystal plane; the path-length difference is given by the difference in the length of the solid arrows. (*b*) The variation in |*A*_Ω_|^2^, along Ω, equation (4), with X = 0 and the *P*_Ω_ included [equation (16*a*)[Disp-formula fd16a]], for various lateral dimensions *L*_*x*_. All the peaks have been normalized and the main graph has been averaged (except the peaks) to show the trend, and the inset indicates the actual complexity of the fringing. (*c*) The extension of (*a*) to multiple planes and how the combination of waves A and C will maintain a phase relationship by allowing *x* to vary, (*d*).

The amplitude *A*_Ω_ at the point P(2θ) (Fig. 4 ▶ *a*) for a beam incident at an angle Ω to a crystal plane is found by deriving the phase difference for different possible paths, which can be represented by a sinc function:
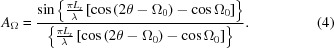
The shape of this profile is a function of the scattering plane lateral dimension in the plane of the incident-beam direction, *L_x_*. The position of the detector is set to detect scattering at an angle 2θ with respect to the incident beam. The maximum reflecting power occurs at Ω = θ and is the specular reflection from a single plane of atoms at this incident angle. *A*_Ω_ is the amplitude recorded at P(2θ) as the crystallite is rocked about an axis normal to the incident and scatter beams (Fig. 4 ▶ *b*).

Within a perfect crystal there will be many parallel planes that scatter as above, and their amplitudes are added taking into account their phase relationships. The maximum amplitude occurs when the phase difference between the different possible paths is zero or *n*π, where *n* is an integer, and when the amplitude *A*_Ω_ is at a maximum. The amplitude combination therefore falls in magnitude as Ω differs from θ, equation (4), and when θ differs from the perfect constructive interference combination of waves, *A*_2θ_. The latter amplitude is analogous to scattering by a diffraction grating, which is the product of the amplitude of a single slit and that from many slits (*e.g.* Jenkins & White, 1957 ▶).

**Figure 5:**
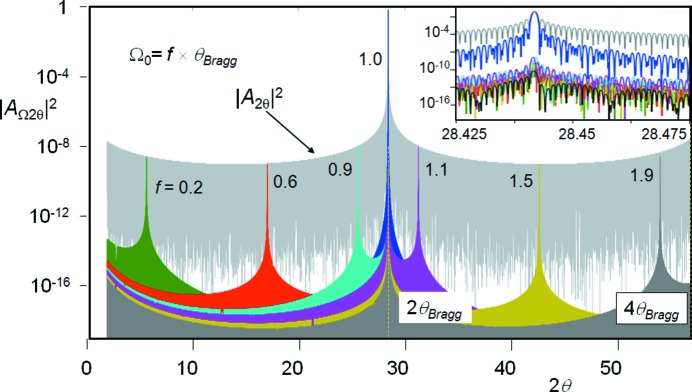
The variation in |*A*_Ω_ *A*_2θ_|^2^, based on a parallelepiped, for different Ω values (given as a fraction *f* of the θ_Bragg_ value), with the inset showing the detail close to the Bragg angle. For each 2θ profile, except at the Bragg condition, there are two peaks in the intensity, at the specular condition when 2θ = 2Ω and when 2θ = 2*θ*_Bragg_. The value of the intensity at the specular peak is given by |*A*_2θ_|^2^. The accumulation of a large number of orientations will add intensity to the tails and to the 2θ_Bragg_ angle, the latter will create enhancement at the Bragg angle, without necessarily being in the Bragg condition.

It is important to show that the scattering from a stack of parallel planes remains in phase when Ω ≠ θ_B_ at the scattering angle 2θ_B_. Fig. 4 ▶(*a*) shows the end point of summing a large number of waves scattered across the surface of a plane; however, the separation between the scattering points, *x* in Fig. 4 ▶(*c*), makes no difference to the end result for *A*_Ω_ provided *x* is small. The trajectories A_0_, B_0_, C_0_ and D_0_ all represent possible paths for a photon, and the combination A_0_A_1_ and C_0_C_1_ and similarly B_0_B_1_ and D_0_D_1_ will always have a value of *x* that will keep them in phase. The path difference Δ for a given *x* is *ab* + *bc* in Fig. 4 ▶(*d*), where 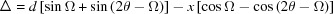
where *x* can be determined by equating Δ = 2*d*sin θ which is the condition when *x* = 0, and must also be satisfied; therefore
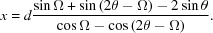
The values of *x*/*d* vary from 0 to a maximum of typically ∼0.1 (for a Bragg angle of 14.2° and 0 < Ω < 28.4°), the phase is only maintained provided *x* < the crystallite dimension or the coherence length. This path difference leads to the amplitude for a stack of parallel planes being
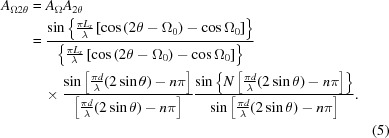
This is a combination of the separation between the planes, *d*, and the number in the stack, *N*, and the incident angle, which is given as Ω_0_ to refer to the case when there is no tilt, X = 0. A plot of 2θ for various Ω_0_ will result in the intensity being concentrated at two positions, when 2θ = 2Ω corresponding to the specular condition expressed in *A*_Ω_, and at 2θ = 2 sin^−1^(*n*λ/2*d*), which is the Bragg angle 2θ_B_ (Fig. 5 ▶). If Ω = θ = θ_B_ the intensity comes to a maximum and represents the Bragg condition.

Hence a powder sample that has a distribution of orientations will create fringes associated with its size and surface shape and an enhancement at 2θ_B_ for each crystallite plane. The magnitude of the size fringes is given by |*A*_2θ_|^2^ since *A*_Ω_ = 1 for a parallelepiped, and the enhancement at 2θ_B_ results from |*A*_Ω_|^2^ since *A*_2θ_ = *N*. Each crystallite with a specific Ω value will create scatter along 2θ that will contribute to the fringing and to 2θ_B_. The contribution to the fringing from many crystallites will be distributed, whereas the enhancements at 2θ_B_ are additive. For a full distribution of Ω values for a parallelepiped the profile in 2θ takes on the form of |*A*_2θ_|^2^ as given in Fig. 5 ▶.

**Figure 6:**
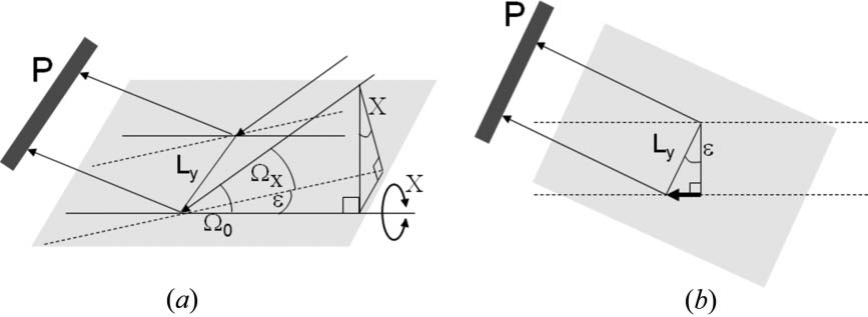
(*a*) gives the path difference from scattering out of the specular plane that results from an inclined plane; the path difference is indicated by the solid arrows in the projection given in (*b*). The beam paths of the maximum intensity for the incident angle Ω_X_ projected on to the diffraction plane follow the dashed lines in (*a*) and (*b*), but the intensity of interest is at P.

The amplitude given above assumes that the crystal plane normal is in the same plane as the source and the detector at P(2θ). However, if this scattering plane is inclined by an angle X, then Ω_0_ is modified to
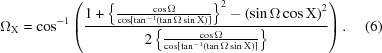
The maximum in the scattered amplitude will now be in the plane of the source and the plane normal and therefore not in the same plane as in Figs. 4 ▶(*a*), 4 ▶(*c*) and 4 ▶(*d*). The amplitude is now modified by this new value for the incident angle and becomes 
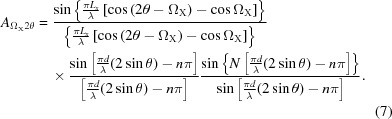
In the diffraction geometry considered, this amplitude refers to that observed by a capture point within the plane containing the source and the scattering plane surface normal. The amplitude contribution at the detector P can be derived from the coherent sum of the various possible beam paths on this inclined scattering plane (Fig. 6 ▶). The lateral dimension of the crystallite, *L_y_*, normal to *L_x_* and *d*, will result in another sinc function that varies with the tilt, X, and is given by 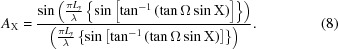
The product of equation (8) with *A*_Ω2θ_, equation (7), will give the amplitude observed at P(2θ) for a scattering plane tilted by X out of the plane containing the source, the centre of the crystallite and the detection point, P, *i.e.* the plane given in Figs. 4 ▶(*a*), 4 ▶(*c*) and 4 ▶(*d*). The full amplitude is given by 
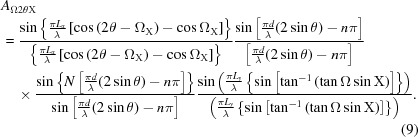
In summary, the amplitude is a function of the interplanar spacing *d*, λ is the wavelength of the X-rays and *n* is an integer, the number of contributing planes, *N*, with dimensions *L_x_*, *L_y_*, and the orientation of these planes to the incident beam, Ω_0_ and X [Ω_X_ is related through equation (6)].

**Figure 7:**
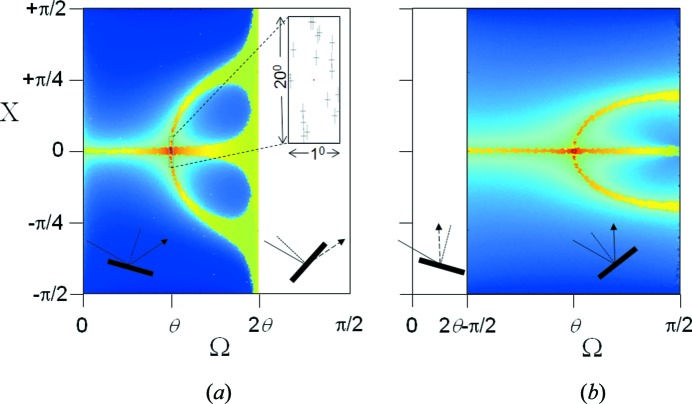
The calculated intensities as Ω and X are varied for a detector at 2θ = 60° (*a*) and 2θ = 110° (*b*) for 10 µm crystallites. The intensity distribution is evaluated by randomly sampling Ω and X positions, 71 262 in (*a*) and 82 362 in (*b*), and integrating over ΔΩ and ΔX associated with the instrument function. The bounds of the instrument function, based on 0.04 radian Soller slits and diffractometer radius of 320 mm, are given by the crosses in the expanded region close to the ‘specular condition’, Ω = θ.

There will be a measurable amplitude distribution everywhere within the bounds 0 < Ω < 2θ (or π − 2θ < Ω < π/2 if 2θ > π/2), −π/2 < X < π/2 and 0 < θ < π/2 (Fig. 7 ▶). The scatter below the plane (Ω > 2θ) and that backscattered (Ω < π − 2θ) is assumed to be weak compared with that reflected forward above the plane, making the calculations faster without changing the subsequent estimates for the mean intensities. The value of *n* in equations (5), (7) and (9) can take on any integer value and is considered briefly. For the case of *n* = 1, Ω ≤ 2θ_B_ since the influence of any diffraction cannot be observed if the maximum is not theoretically accessible. If *n* > 1, harmonic components of *hkl* would be expected, *i.e.* path lengths at multiples of the wavelength; however, the enhancement is weaker and their magnitude is dependent on high levels of perfection, which makes it less relevant to powder diffraction. Ω can take on a maximum value of π/2, before the scattering is directed towards −2θ, and for Ω > π the scattering will not be from the (*hkl*) plane but from (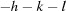). Similarly for Ω < 0, the scattering will only come from the (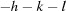) plane towards −2θ and switches to the +2θ for Ω < −π/2. The regions incorporating −π < X < −π/2, π/2 < X < π relate to scattering from the underside of the planes and correspond to the reflection 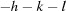. Fig. 7 ▶ can be considered as the intensity distribution from a single crystallite with the detector set at 2θ. Therefore, there will be scattering captured by the detector from this single crystallite as it is tilted in X and rotated in Ω.

The locus and intensity of a Debye–Scherrer ring are represented in Fig. 7 ▶ as a line at a constant Ω. Ω represents the maximum incident angle on the crystal plane for a specific orientation. As the detection point is moved around the ring, this is equivalent to rotating in X. However, this change in X results in a new incident angle, Ω_X_, for that detection point. The locus of stronger intensity where X ≠ 0, for example associated with the specular (or Bragg peak), occurs where Ω_X_ = θ that can only be accessed by increasing Ω. What this means is that a crystallite will contribute intensity to the Debye–Scherrer ring over ± π/2 that peaks at X = 0 if Ω < θ and at ±X and X = 0 if Ω > θ. For Ω > θ the characteristic three spots should indicate the deviation of Ω from θ.

The structure factor described in equation (2) should be considered more carefully. The structure factor is better described as the scattering power, since it represents the integral of all the scattering from a plane with indices *hkl*. The scattering power is assumed, based on pure ‘Bragg scattering’, to exist at the Bragg angle, with some small allowance for the peak broadening in conventional powder diffraction theory, *i.e.* the structure-factor influence is smeared. It is important to differentiate between the intensity measured and to what it is assigned, since the scattering power [the full integral of equation (2)] is the sum of all the possible scattering from the *hkl* plane. The scattering power corresponds to the sum of all the amplitude contributions within the bounds given above, integrated over the accessible range of 2θ. Therefore the measured intensity does not necessarily relate simply to a representative estimate of the scattering power. This is discussed further in the next section.

**Figure 8:**
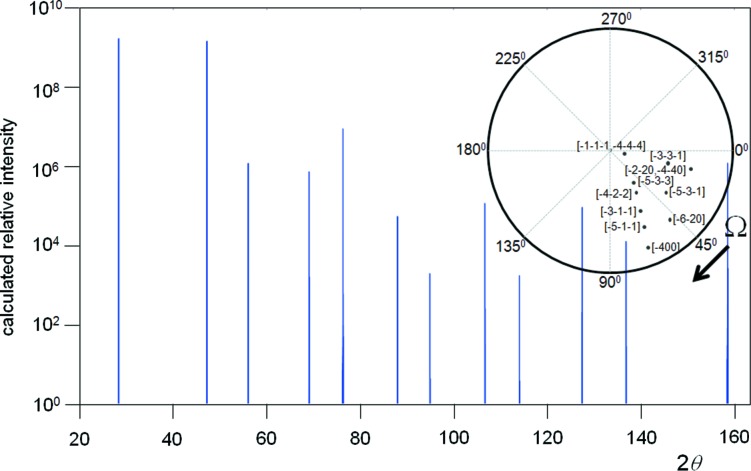
The calculated scattering captured by a linear detector with no axial width from a single Si crystallite fixed at one orientation, which gives an indication of how the powder diffraction pattern is created. This pattern was one that gave the highest number of reflections, chosen from a randomly orientated set of ten crystallites. Typically the number can be anywhere between 0 and 12; the latter is the full complement of unique peak positions out of the 246 reflections. The reflections that were captured in this angular range appear in the right-hand lower quadrant and represent the ranges 0 (or π − 2θ) < Ω < 2θ (or π/2), −π/2 < X < π/2 and for 0 < 2θ < π (provided 2θ < 4θ_B_).

Clearly if this scattering can be observed from a specific *hkl* reflection that fits these bounds, then any crystal plane orientation that fits these bounds will also produce scattering that will have a maximum intensity capture for detection at its 2θ = 2 sin^−1^(*n*λ/2*d*). A single orientation of a single crystallite can therefore produce scattering from a large range of *hkl* reflections. Hence the full scattering pattern from a polycrystalline powder will emerge from very few crystallites, although the intensities will be very variable until a reasonable statistical sample is obtained. As an example, a simulation of the scattering from a single Si 10 µm crystallite is given in Fig. 8 ▶, where all the *hkl* reflections that lie within the bounds defined above will contribute to the detected signal. This particular example was one out of seven randomly chosen orientations, which included on average 6.3 reflections, ranging from 3 to 12 (0 reflections have been recorded but not in this set).

It is recognized that the crystallite has been defined by three dimensions, *L_x_*, *L_y_* and *L_z_*, which represent a parallelepiped, and not the full shape. This is discussed in the section on dynamical scattering effects and whatever is used will necessarily be an assumption. The rotating parallelepiped given in this description will result in enhanced intensity normal to the surface planes evident in Fig. 5 ▶ as |*A*_2θ_|^2^ and in Fig. 7 ▶ as |*A*_Ω_|^2^ at X = 0. The introduction of various shapes creates a different distribution of fringing, but the enhancement at 2θ_B_ is still present (Anderson & Fewster, unpublished work).

These amplitudes and these angular coordinates are now mapped onto the diffractometer to obtain the summation ranges in equation (9) and from that the relevant intensities can be determined.

## 6. The new intensity formula   

The resultant intensity captured by the detector at P(2θ) is then the integral of all the contributions that can pass from the source to the detector *via* the crystallite. The first of the following equations represents the condition when the coherence of the X-rays exists over large areas of the source and at the detector, equation (10). This is unlikely in a typical powder diffraction experiment, and so the coherent sum should be over a smaller region defined by the X-ray coherence length: this is typically in the region of 4 µm corresponding to an angular acceptance of 0.001° within the minimum sampled region of 0.01° × 2.3° (Fig. 7 ▶). Therefore the practical experiment is best represented by the second equation (11). 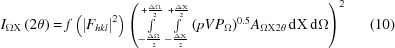


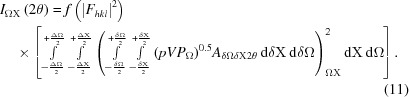
ΔΩ represents the angular range accepted by the crystallite, ΔX is the angular range of possible paths of the beams in the axial plane (typically defined by Soller slits) and is discussed below. δΩ and δX represent the region that is coherently linked (effectively the region of probability that a photon can occupy). The various parameters *p*, *V* and *P*_Ω_ will be discussed below. 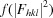 is a function related to the scattering power (structure factor).

The parameter *p* is the polarization factor, which takes into account the changes in the two orthogonal components of the electric field of the electromagnetic wave, which occurs on scattering. From geometry and for the general case when a monochromator is used, *p* is given by
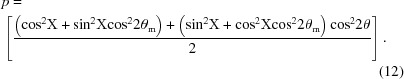
θ_m_ is the Bragg angle for the monochromator. This polarization term is associated with the scattering peak and, therefore, the σ component (first bracketed term) and π component (second bracketed term) are projected onto the reflecting plane for the specular peak. If there is no monochromator and the incident beam is circularly polarized then this reduces to the familiar form with 2θ_m_ = 0, *i.e.* 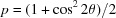.

**Figure 9:**
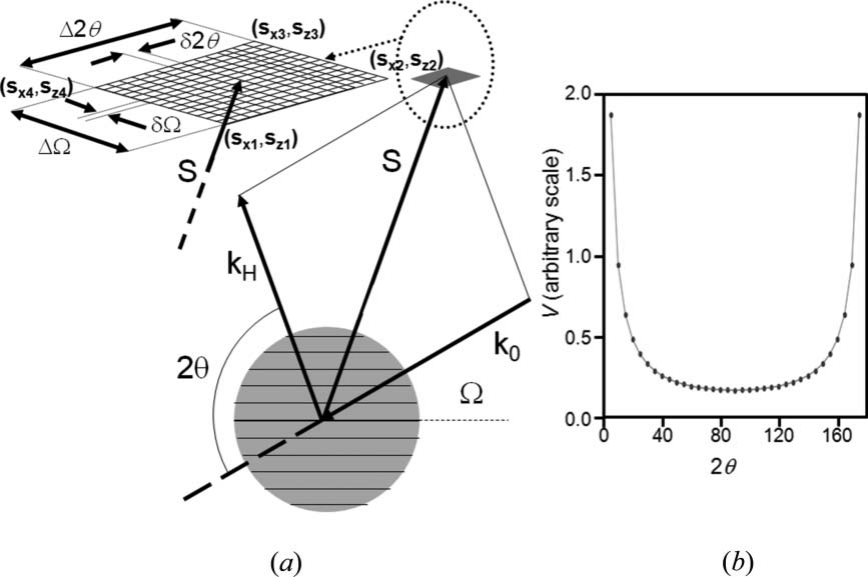
(*a*) The instrument capture area for a spherical crystallite in the plane normal to the crystal plane tilt, *i.e.* for X = 0. The integration steps, in Ω and 2θ, are inclined with corners of the area defined by (*s_xi_*, *s_zi_*), where *i* = 1, 2, 3, 4. (*b*) gives the variation in this capture area as a function of 2θ (points; calculated from random values of Ω) and the Lorentz function 1/sin 2θ (line). **S** is the diffraction vector that defines the area in reciprocal space for an incident-beam vector of **k**_0_ and a detected-beam direction defined by the vector **k**_H_.

**Figure 10:**
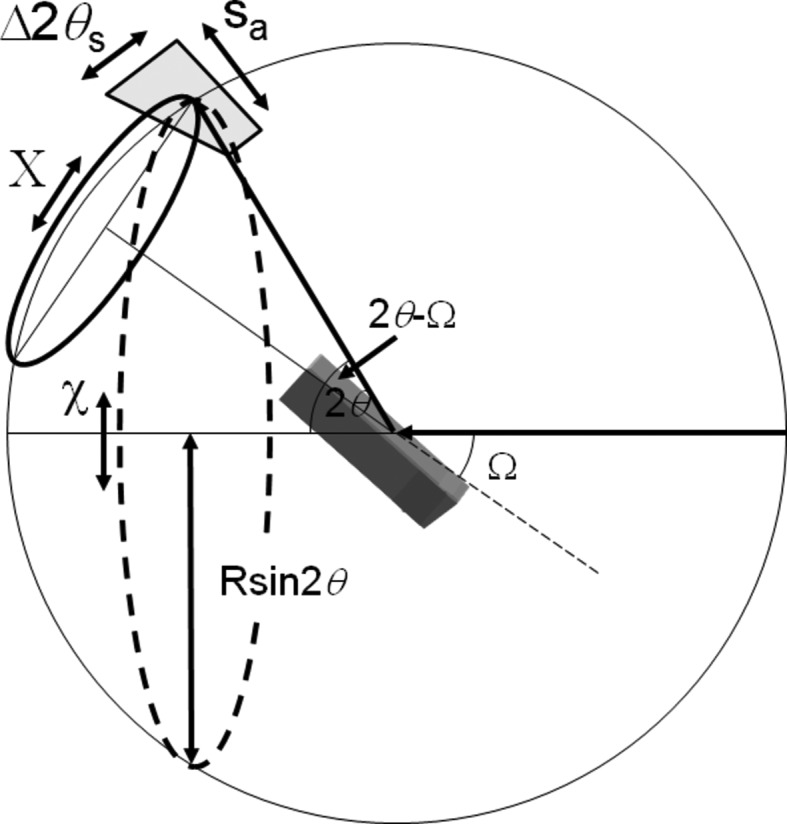
The capture-volume variation with incident angle Ω and scattering angle 2θ. The detector slit dimension is defined by Δ2θ_s_ in the diffractometer plane, and the slit dimension, or Soller slit, normal to the diffractometer plane, *s*_a_. As the angle 2θ–Ω is reduced the range in X, *i.e.* ΔX, becomes large. The Debye–Scherrer ring is given by the variation in χ (dashed line).

The parameter *V* is the volume over which the intensity is captured in the experiment and is fixed in this case by the size of the rectangular receiving slit or detector to which the intensity is assigned. However, the intensity reaching this slit is determined by the region in reciprocal space which has bounds defined by ΔΩ, ΔX and Δ2θ_s_, where Δ2θ_s_ is the angular acceptance of the detector slit. The two bounds ΔΩ and Δ2θ_s_ are by definition orthogonal to ΔX. The contribution from the area bounded by ΔΩ and Δ2θ_s_ is given in Fig. 9 ▶(*a*), which is bound by reciprocal-space coordinates (*s*_*xi*_, *s*_*zi*_) where
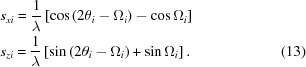
The area in reciprocal space can be determined numerically by Heron’s method (Heath, 1921 ▶) and the results are plotted in Fig. 9 ▶(*b*). The evaluated capture area in reciprocal space is overlaid with the conventional 1/sin (2θ) Lorentz factor and clearly this derivation gives exactly the same results. What is important here is that the derived capture volume is completely general and is not reliant on a diffraction peak moving in and out of the Bragg condition as in the conventional definition of the Lorentz factor. Also it is independent of the incident angle Ω: the Ω values plotted in Fig. 9 ▶(*b*) are selected randomly between 0 and 2θ. The parameter *V* is therefore given by 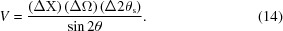
The contribution, ΔX, is normal to the Ω/2θ plane and its axis lies in the diffractometer plane, and will now be considered. The range of tilt of the crystal plane ΔX that can capture intensity will vary depending on the angle of the tilt axis to the data-collection point, 2θ − Ω (Fig. 10 ▶). When the angle (2θ − Ω) is small, the angular acceptance region in reciprocal space, defined by ΔX, will accept intensity over a wide range of tilt X of the sample. Hence this large reciprocal volume will contribute to a small angular range in ΔX. Therefore the intensity at a specific Ω and X should be the sum of all the contributions over the region of diffraction space that can be captured. The spread in X over which this sum should include is given by 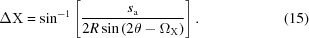

*R* is the sample-to-slit distance and *s*_a_ is the effective axial slit width, *i.e.* the equivalent lateral dimension that would accept scattering from a single crystallite within the sample. If (2θ − Ω) < sin^−1^[*s*_a_/(2*R*)] then the captured scattering in X includes the full bounds: −π/2 < X < π/2.

Another factor influencing the final intensity is the incident-beam projection onto the scattering planes. Clearly, at low incident angles, the proportion of the incident-beam flux that an atomic plane can scatter is small and increases to a maximum at normal incidence. The incident beam of dimension *B*_*x*_ will interact with a projection given as 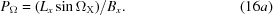
This assumes that the crystallite under analysis is completely bathed in the incident beam. As an example when Ω_X_ is small the cross section of the crystallite is small and the scattered intensity is reduced. For a crystallite plane projection larger than the incident beam, the total flux available for scattering is unchanged so 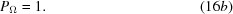
Equations (10) and (11) represent the intensity at a specific coordinate and therefore the full intensity at 2θ from a large number of crystallites *N* with a random distribution of orientations is given by 
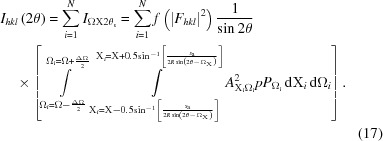
This summation will give the intensity at 2θ from a specific set of *N* *hkl* planes that have orientations with respect to the incoming X-rays and detector slit defined by Ω and X. This equation is applicable to a single crystal or crystallite (*N* = 1) and gives the integrated intensity captured at the detector at position 2θ, associated with a specific set of crystal planes in an orientation defined by Ω and X. If, however, the sample is composed of a large number of randomly orientated crystallites then the accumulated intensity at 2θ can be considered as the sum of this intensity ‘sheet’ in Ω and X with a ‘thickness’ of 2θ_s_. The influence of the detector slit (2θ_s_) can be approximated by a convolution if it is assumed that a large number of crystallites contribute or the slit is narrow. If there are only a few crystallites, then the contributions from the intensity sheet are correspondingly less and may not give stable reliable estimates of the intensity, since the very nature of the distribution is that it is heavy tailed, *i.e.* similar to a log-normal distribution with a truncation point intensity at Ω = θ.

*I*(2θ) is the intensity captured by the detector at 2θ, so to obtain the intensity along the whole profile, the amplitude sheet should be calculated for each 2θ step value, although as will be discussed in the next section this can be simplified with a good distribution of crystallite orientations. This equation indicates that, when exposed to the incident beam, each crystal plane of each crystallite scatters intensity over much of the region above each crystal plane. It is the summation of all the contributions from all the *hkl* reflections that results in the final profile. Since all crystallites are able to contribute intensity almost everywhere, they are not required to be orientated to the Bragg condition for intensity to reach the detector. The profile along 2θ is also not confined to the region close to the Bragg condition, so this equation will determine the ‘background’ intensities and full peak profiles *etc*.

## 7. The scattering power of a reflection   

**Figure 11:**
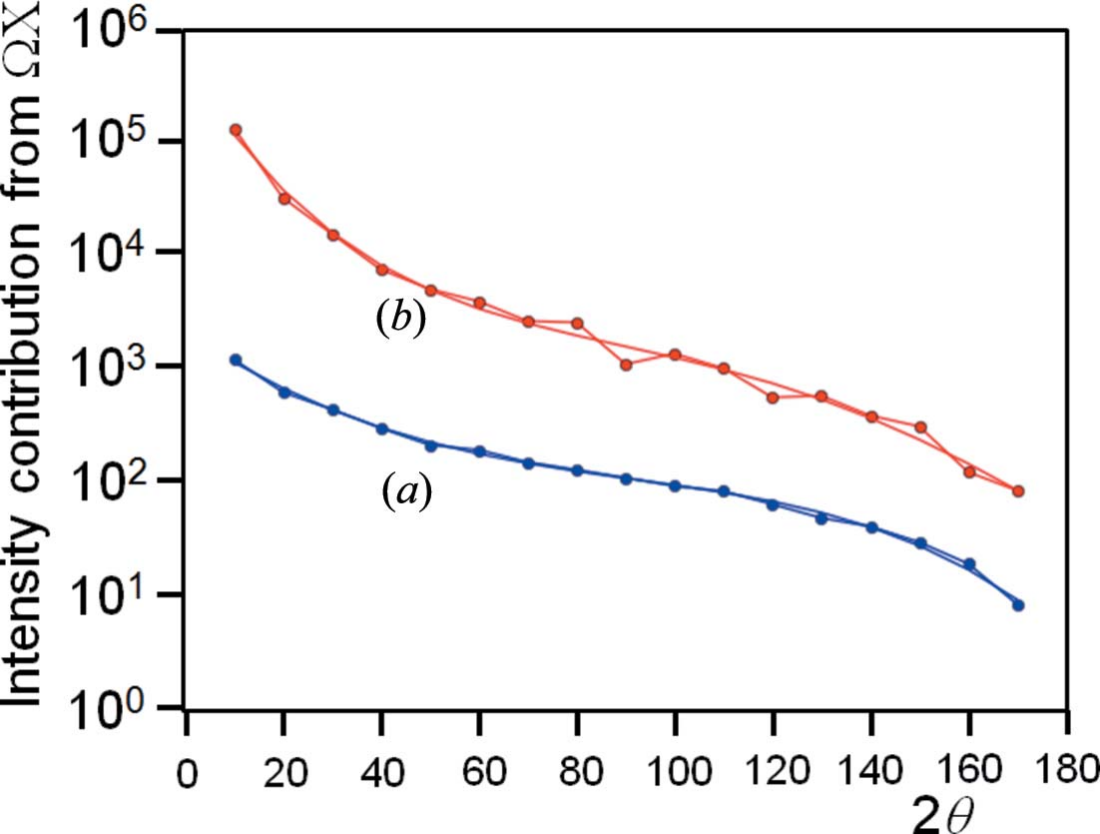
(*a*) The calculated dispersion of the scattering power in Ω and X as a function of 2θ (effectively the integral of Fig. 7 ▶ without oversampling from axial divergence). (*b*) The calculated integrated intensity in Ω and X as a function of 2θ with the variable axial divergence limited by Soller slits as in an experiment (equivalent to the integral of Fig. 7 ▶ with oversampling).

The function 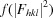 will now be discussed. The scattering power or structure factor 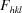 is the amplitude distributed throughout the range of scattering in 2θ, Ω and X, that fits within the bounds defined earlier for the plane *hkl*, and the intensity measured will be governed by kinematical or dynamical theory. Clearly, the scattering is distributed in X and Ω, as in Fig. 7 ▶, and throughout 2θ, *i.e.* the bounds in Ω are from 0 to 2θ_B_ or (π − 2θ_B_) to π/2 if 2θ_B_ > π/2, and for X −π/2 < X < π/2, and for 2θ 0 to 4θ_B_ or 0 to π if 2θ_B_ > π/2. Thus there is a three-dimensional distribution of scattering, the sum of which is the total intensity associated with the scattering power. The dispersion of this scattering power is 
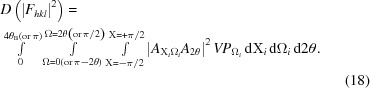
This integral was evaluated by sampling, using the following expression: 
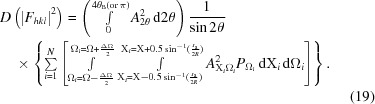
However, in an experiment, the evaluation of the integrated intensity from a set of crystallographic planes usually only captures the intensity close to the ‘Bragg condition’, assuming that the background contributes no additional scattering associated with the structure information. The integrated intensity is then usually related to |*F_hkl_*|^2^ in the kinematical approximation. However, this assumes that the whole of *F_hkl_* is captured, which is not correct. The factor *f* is the ratio of the integrated intensity over the measured limits, compared to the full integral, equation (19). This is also the case for single crystals and will be discussed later. From the last section it can be seen that, in the powder diffraction experiment, when the distribution of orientations is very large, the captured volume of the scattering in Ω and X will include the whole distribution. However, because the instrument capture varies with ΔX, equation (15), the data collection will oversample, so the intensity is overestimated depending on Ω and 2θ. The factor *f* will therefore include two components: the limited capture range for obtaining the integrated intensity and the oversampling due to the axial divergence: 
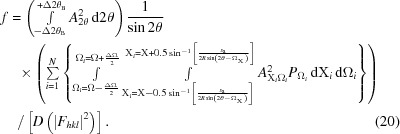
The ΩX map, Fig. 7 ▶, represents the distribution of intensity at a specific 2θ for a specific *hkl* reflection. The intensity everywhere on the ΩX map represents the residual contribution to the specular, and therefore also Bragg, contribution. The overall resultant observed intensity is then the phase sum at this specific 2θ. For example, at the Bragg condition, all the contributions scatter in phase and further from this the differing phase contributions create an interference pattern. The integral, equation (20), also represents the distribution of the scattering power in Ω and X at a specific 2θ, which varies as in Fig. 11 ▶(*a*) for a 10 µm crystallite. This, combined with the contribution along 2θ, will give the full dispersion of *F_hkl_*. This gives the intensity distribution values over three dimensions.

By modifying the number of crystallites *N*, in equation (17), it is possible to estimate the variability of the intensities and the proportion of the scattering that can be associated with the structure factor.

## 8. Dynamical scattering effects   

**Figure 12:**
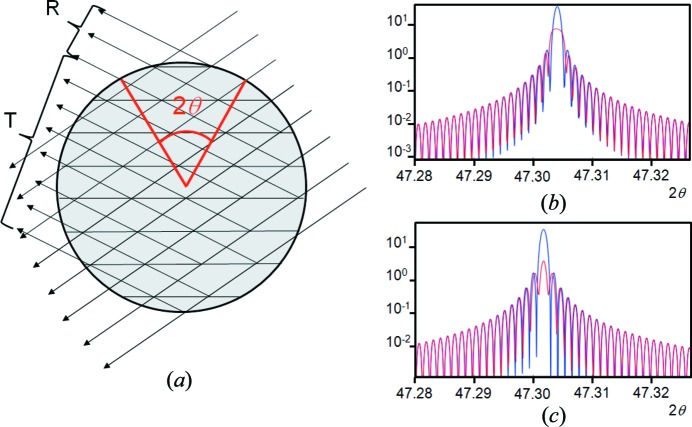
(*a*) The simplified basis for establishing the proportion of reflected (R) and transmitted (T) waves for a spherical crystallite. (*b*) The profile based on dynamical theory in reflection mode (red, lower central peak) compared with kinematical theory for a flat plate sample, and (*c*) the profile for the transmission case.

The absorption and losses through scattering are not included in the equation for the amplitude *A*_2θ_, equation (9). For most structures, absorption should be included, and for those materials that scatter strongly then dynamical theory must be considered. In general, a crystallite will be completely immersed in the beam and all path lengths for all reflections will be similar; therefore, absorption should be similar for all reflections and will act as an overall scale factor. Kinematical theory presented here assumes that the crystallite is composed of a series of scattering centres, and that doubly scattered waves, leading to a reduction of the refracted wave, have little effect (‘primary extinction’). Dynamical theory considers all these interactions but is easily disrupted by defects and distortions (*e.g.* Note 1[Statement note1]). Dynamical theory is a wavefield approach and depends on the boundary conditions as the wave enters and exits the crystallite. Without modelling the detailed shape of each crystallite, a few assumptions need to be made. If the crystallite is assumed to be spherical, then the intensity weighted mean of contributions in the reflection mode and transmission mode can be estimated from geometry (Hart *et al.*, 1988 ▶). The differentiation between the two modes is that in reflection mode only one branch of the dispersion surface is excited, whereas in transmission mode two branches of the dispersion surface are excited. To excite only one branch of the dispersion surface, the surface normals, for the entrance point of the incident wavefield and the exit point for the reflected wavefield, have to be within (π/2 − θ) of the diffraction vector (crystal plane normal) (Fig. 12 ▶ *a*). The area of the hemisphere illuminated by the beams that satisfy the reflection mode is (4π*r* ^2^2θ)/2π, and therefore the fraction that satisfies the reflection mode is 2θ/π. This fraction was used to proportion the reflection-mode and transmission-mode contributions.

These dynamical effects are most pronounced at high intensity; the forward double-diffracted beam is strong enough to moderate the incident refracted beam, and the diffracted beam removes a significant proportion of the incident refracted beam. This also is only significant close to the Bragg condition. Outside this region, it is coincident with kinematical theory and therefore dynamical theory only needs to be applied to scattering where Ω ∼ θ_B_.

The amplitude *A*_2θ_ based on dynamical theory in reflection mode is 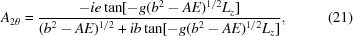
where 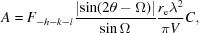


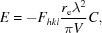


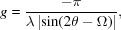

*r*_e_ is the electron radius, λ is the X-ray wavelength and *C* is the polarization factor that is 1 or cos 2θ. The parameter *b* is given by 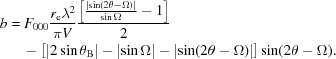
These are the formulae for the two-beam plane-wave dynamical theory with two tie points, *e.g.* Fewster (2003 ▶). This calculation has to be performed for both polarizations and added. If a monochromator is used then *C* takes on values of 1 and cos 2θ_m_cos 2θ. θ_B_ is the Bragg angle, specific to the set of planes calculated, and θ_m_ is the Bragg angle for the monochromator. If the incident wave impinges on the crystal surface, or crystal plane below the critical angle for total external reflection (typically ∼ 0.2°), then the intensity scattered into 2θ, provided 2θ > 2× critical angle, should be ∼ zero, although the contribution is very weak from the projection effect, equation (16*a*)[Disp-formula fd16a], so neglecting this is not serious. Any wave emerging from the exit surface, or a crystal plane below the critical angle (*i.e.* for the latter 2θ − Ω < critical angle), may not emerge as well defined scattering, depending on the shape of the exit surface. The latter intensity contribution is insignificant compared to the instrumental effects of equation (15). Therefore the intensity contributions from within the critical angle are set to zero in the ‘sinc’ function and dynamical models, since this is the closest approximation without modelling the crystal shape.

A more fundamental approach to the dynamical model based on the atomic positions and scattering factors, not requiring structure factors or Bragg angles, can yield the amplitudes by slicing the crystallite into very thin parallel lamellae (∼0.001 nm) (Holý & Fewster, 2008 ▶). However, the calculation time is increased substantially and the results are essentially unchanged from those calculated here, unless the crystallites are very small, in which case the scattering is well represented by kinematical theory or the Debye formula (Debye, 1915 ▶). This plane-wave dynamical theory is still an approximation for many experiments and spherical-wave theory would be less of an approximation; however, within all the other assumptions regarding crystallite shape and the possible lens nature (adding to some divergence) of the scattered beam, the dynamical model used here is sufficient.

The amplitude *A*_2θ_ in transmission mode is based on Zachariasen (1945 ▶) and is given by 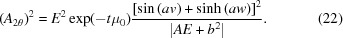
The parameters not given above are 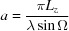


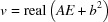


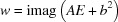


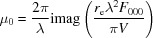


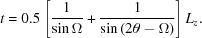
The parameter μ_0_ is the average (kinematical) linear absorption coefficient, which is a valid assumption for the case studied here. For crystals greater than the absorption depth (65 µm for Si) the separate absorption coefficients should be considered; these are associated with the two branches of the dispersion surface and their two polarization states, leading to different values. By reducing the value of *F_−h−k−l_* that corresponds to the scattering from the underside of the reflecting planes, which interferes with the refracted wave, then the profile becomes indistinguishable from kinematical theory and the sinc function, for both the reflection and transmission modes. The amplitudes based on dynamical theory, for both transmission and reflection modes, have a similar dependence, *i.e.* the dynamical effects reduce the low 2θ and intense reflections. The dynamical effects can be seen by calculating the profile from an 8 µm Si parallel-sided flat plate, for each reflection, and comparing it with kinematical theory (Fig. 12 ▶ *b* for reflection, and Fig. 12 ▶ *c* for transmission). The magnitude of the dynamical effects can be reduced and will have a dramatic effect on the calculated intensities. The most influential variable, though, is to change the kinematical/dynamical proportions by changing the contribution of *F_−h−k−l_*. It can be shown that the dynamical impact, defined by 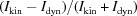, is less than 6% for Si crystallite dimensions ∼1 µm, giving some guidance for the influence. This ratio gives an indication of the largest average length scale over which defects can be separated, without needing to invoke dynamical theory in Si, based on analysis of just the Bragg condition. The presence of defects has an additional effect of introducing diffuse scatter, which is discussed later.

## 9. The calculation of the scattering profile   

Because of the widespread use of the Bragg–Brentano diffraction geometry, the emphasis here will be on calculations and results from this configuration. The calculations are lengthy and full use is made of parallel computing. In the ideal case, the intensity distribution in X and Ω should be calculated for each scattering plane *hkl* and at each step in 2θ. In this way, the full multiplicity can be incorporated, as in Fig. 8 ▶, and also the trade-off between numbers of crystallites and flux can be included, equations (23) and (24). To overcome the limitations in computing power, a calibration curve was calculated at various 2θ values, assuming that this represented the variation in the captured intensity with scattering angle. The captured intensity was obtained by uniform sampling in X and Ω, and superimposing the instrument capture volume ΔΩΔX, and integrating [ΔΩ is defined by the focus and crystallite size and ΔX is a function of (2θ − Ω)]. This procedure is repeated until the intensity distributions start to converge, *i.e.* most crystallite orientations have been explored and captured. This method is applicable to randomly orientated crystallites. For samples with texture or preferred orientation, a prior probability should be imposed (rather than uniform sampling), and this would ideally need to be done for each set of *hkl* planes, so that the multiplicity is incorporated naturally, as in Fig. 8 ▶. Early attempts at this latter approach proved prohibitive in time. For the calculation here, where it is assumed that no preferred orientation exists, the multiplicity has been incorporated as a multiplicative factor when applying it to the specific reflection concerned. The calculations are performed in 64-bit Python with heavy use of vectorization with NumPy and Parallel Python using up to 32 cores; this was far from optimal but appropriate for testing the theory at this stage. The results from obtaining a calibration curve are now considered.

### 9.1. Intensity variations associated with the Bragg–Brentano geometry   

The above derivation assumes that the number of crystallites illuminated is constant with respect to the sample as a whole. In the para-focusing geometries, *e.g.* Bragg–Brentano geometry, this is not the case, and the number of crystallites illuminated changes with incident angle according to
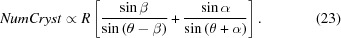
Here, α and β are the divergence angles of the beam below and above, respectively, the source to the goniometer axis, which makes an angle θ to the sample surface. The number of contributing crystallites is increased at low θ angles; however, the distribution of X-ray flux is reduced according to
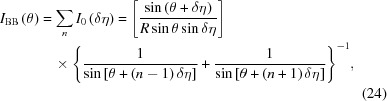
where δη is a small constant angular increment in the divergence. This has a similar inverse form to equation (23), and this is why the assumption that the two completely different geometries, Bragg–Brentano and Debye–Scherrer, can use a similar formula in conventional theory. However, there are small differences, *e.g.* the flux varies across the sample, and this is most pronounced for smaller radii and larger samples. For a fully illuminated 10 mm sample with a 240 mm goniometer radius, the intensity variation across the surface is 1.8% and 0.45% at 28° and 88° 2θ, respectively. Perhaps a more important point here, though, is that if the *NumCryst*/flux ratio is roughly constant (this varies by 10^−4^ from 28° to 160° 2θ), then this would suggest that the diffraction profile in the Bragg–Brentano geometry would have increasing unreliability with increasing 2θ as the distribution of crystallites satisfying the Bragg condition is reduced, if the conventional theory were to be correct. For the calculation in the new theory, this larger number of illuminated crystallites (and reduced flux per crystallite) can be included.

### 9.2. The intensity associated with Bragg and non-Bragg   

It is important now to assess the contribution to the captured intensity from crystallites in random orientations compared with those contributing at the Bragg condition. This evaluation can be achieved by uniformly sampling over Ω and X, then integrating over the vicinity to emulate the angular acceptance of the crystallite to the divergence in both directions. This will also give an estimate of the proportion of the intensity associated with the Bragg condition, compared with that from non-Bragg, for a stationary sample: essentially, the intensity distribution for a specific experimental configuration. The variation in the intensity summation for each ‘sheet’ at a representative set of 2θ values is given in Fig. 11 ▶(*b*). These calculations assume that the crystallites are small compared with the size of the incident beam, and also include the probability, Pr, of capturing significant scattering over the calculation range, *i.e.*

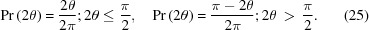
Initially, uniform sampling was carried out whilst monitoring the convergence and variance of the mean intensity; however, because the Bragg condition is such a rare event (*i.e.* the central specular condition Ω = θ and X = 0 in Figs. 4 ▶ *b* and 7 ▶) a vast number of crystallites are required to explore it and to reduce the variability. After many millions of samples, with an integration of the instrument capture at each position, the solution is far from converged, and since this cannot easily be achieved analytically, an integration method based on the idea of importance sampling was used. The map was separated into areas and sampled to achieve a mean; the mean was scaled to the dimensions of the area. This allowed convergence at a far faster rate in a practicable timescale.

**Figure 13:**
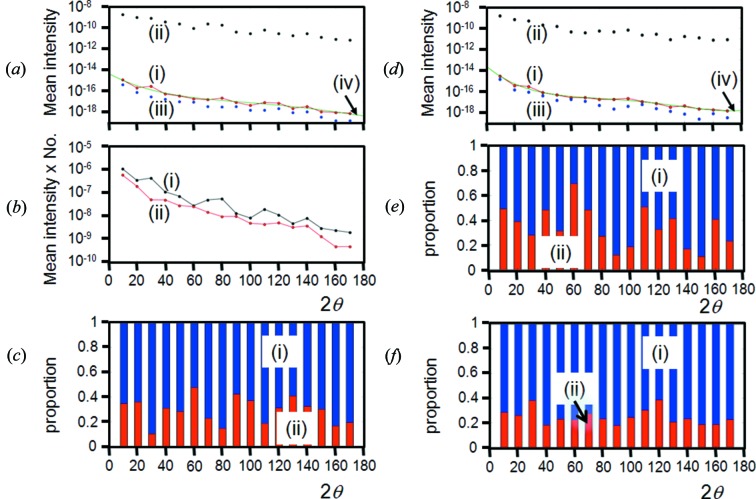
(*a*) The mean calculated intensity as a function of 2θ: (i) overall mean from all contributions, (ii) only those contributions that capture the ‘specular’ peak, (iii) the mean intensity of those from the ‘non-specular’ and (iv) the best fit profile through the overall mean intensity. (*b*) are the mean intensities × numbers for the ‘specular’ (i) and the ‘non-specular’ (ii). (*c*) gives the ratio of the proportions of ‘specular’ (i) and ‘non-specular’ (ii) to the overall intensity. (*d*) and (*e*) are similar to (*a*) and (*c*), except the calculations are based on 3 µm crystallites. (*f*) compares directly with (*c*), except the calculation is for 10 µm crystallites with defects, *i.e.* diffuse scattering is included.

The variation in the intensity captured as a function of 2θ is given in (i) of Fig. 13 ▶(*a*). This is a calibration curve that can be applied to each intensity value at 2θ. If the specular peak corresponds to the appropriate 2θ_B_ coherent Bragg condition, then the mean intensity value may not follow the kinematical theory and so it is useful to separate the ‘Bragg’ and ‘non-Bragg’ contributions, (ii) and (iii) in Fig. 13 ▶(*a*). The Bragg condition is assumed to be satisfied whenever the Bragg peak appears in the accepted divergence of the instrument. Hence provided the incident beam and the scattered beam exist above the scattering plane and they are on the opposite sides of the plane normal, then the ‘non-Bragg’ condition is satisfied, whereas the ‘Bragg’ condition only exists under exacting requirements. This is re-expressed in Fig. 13 ▶(*b*), by plotting mean values of the specular and non-specular, multiplied by their numbers to give the effective intensity contributions from each. The intensity measured in the specular (and therefore the Bragg condition) is composed of ∼30% ‘non-Bragg’ and ∼70% ‘Bragg’, with a standard deviation of 10% for these values for 10 µm crystallites (Fig. 13 ▶ *c*). The calculation is based on sampling the equivalent of 153 000 000 events at 2θ = 10°, to a maximum of 1 374 000 000 at 90°. With more sampling this standard deviation will reduce; however, this does give an estimate of the proportions of the scattering attributed to ‘Bragg’ and ‘non-Bragg’, in the limit of a very large number of crystallites (Fig. 13 ▶ *c*). A typical experiment may capture none, or very few Bragg events; however, the mean intensity will only be displaced by a small amount [Fig. 13 ▶ *a* (i) compared with (iii)]. This indicates that the impact of capturing Bragg events will not have a large effect on the measured intensity. Also, if the number of Bragg events changes roughly in proportion across the 2θ range, then the relative intensities will be unchanged. These calculations were repeated for 3 µm crystallites (Figs. 13 ▶ *d* and 13 ▶ *e*), and for 10 µm crystallites that contained defects (*i.e.* diffuse scattering was introduced) (Fig. 13 ▶ *f*). The introduction of diffuse scattering caused no observable difference in the ratio. For 3 µm crystallites, the ratio is changed to 65 (15)% for the ‘specular’ contribution, suggesting that the ‘specular’ and therefore ‘Bragg’ contributions are less significant for small crystallites.

The importance sampling method used does reduce the variability of the specular, and increase the variability of the non-specular, compared with a totally random sampling method. This is because the density of samples is much higher in the former and would require a comparable number of crystallites to give rise to the number of events quoted, and much lower in the latter. This explains why the profiles are not perfectly smooth [Figs. 13 ▶ *a* (iii) and 13 ▶ *d* (iii)]. It was impractical to sample this equivalent number of events. In fact, the equivalent number of events quoted is derived from the sampling density within 0.5° of the specular position, whereas elsewhere the sampling is sparser and scaled. The total number of samples for each of the 17 2θ values was 81 000.

The overall ‘converged’ mean intensity [Fig. 13 ▶ *a* (iv)] [and Fig. 13 ▶ *d* (iv)] was obtained by fitting a fourth-order polynomial to 13 ▶ *a* (i) [and 13 ▶ *d* (i)], once sufficient numbers of calculations had been completed and the profiles had stabilized. This, as mentioned earlier, represents the situation with a large number of randomly orientated crystallites.

The overall intensity captured during an experiment at the ‘Bragg position’ (or ‘specular position’) is the product of the number of contributions and mean intensity associated with the ‘Bragg condition’ (or ‘specular condition’), plus the product of the number of contributions and mean intensity associated with the ‘non-Bragg condition’ (or ‘non-specular condition’): 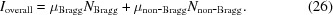
These mean ‘Bragg’, ‘non-Bragg’ and ‘overall’ intensity contributions are given in Fig. 13 ▶(*a*), where a significant difference can be seen in the ‘Bragg’ and ‘non-Bragg’ means. However, the product of the number of contributors of ‘Bragg’ and ‘non-Bragg’ with their respective mean intensities result in very similar values (Fig. 13 ▶ *b*). The result of this is that the overall mean intensity is very similar to the mean intensities of the ‘non-Bragg’ contributions, Figs. 13 ▶(*a*) (i) and 13 ▶(*a*) (iii), and 13 ▶(*d*) (i) and 13 ▶(*d*) (iii).

The calculated mean intensities converged to the values given in Fig. 13 ▶(*a*) (i), which could then be fitted with a fourth-order polynomial as a function of 2θ, Fig. 13 ▶(*a*) (iv) for 10 µm crystallites, 13 ▶(*d*) (iv) for 3 µm crystallites and Fig. 11 ▶(*b*) for 10 µm crystallites with defects. The profiles all follow the same shape. If we assume that this fit represents the converged mean intensities, then the overall variability expected from the experimental intensities would be related to the number of contributions of ‘Bragg’ (‘specular’) and ‘non-Bragg’ (‘non-specular’) and each will follow a Poisson distribution, *i.e.* σ = *N* ^1/2^. The overall variance for the two contributions is therefore given by
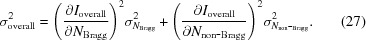
Combining equations (26) and (27) will give 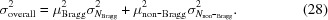
The expected reliability of the intensity measurements in an experiment can be expressed as σ_overall_/*I*_overall_, by using equations (26) and (28), and is given by 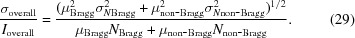
The conventional theory is based on only the first term in both the numerator and denominator, and this will be referred to as the ‘Bragg’ σ/*I*.

### 9.3. The ratio of Bragg to non-Bragg events   

The above calculation gives estimates for the mean intensities associated with the ‘Bragg condition’ (or ‘specular condition’) and the ‘non-Bragg condition’ (or ‘non-specular condition’), for any experimental configuration. To estimate the level of reliability, as σ/*I*, the number of ‘Bragg’ and ‘non-Bragg’ events is required, equation (29). Since any experiment may have different numbers of crystallites, it is more helpful to calculate the ratio of the number of events, which is more universal. The ratio of ‘specular’ to ‘non-specular’ events associated with a particular experimental configuration can be estimated as in the following two sections: these determine the ratio directly, whereas the above calculations include intensity estimations as well, which is considerably slower to calculate. Because these calculations require a large number of repeats, the results presented here are given as a range of both calculations, to give an indication of typical values for the instrument configurations described later.

For the same number of calculated events across all 2θ values the number of Bragg events at 10° is approximately twice that for 2θ > 30°, whereas the intermediate value at 20° is ∼70% more than for 2θ > 30°. The ‘specular’ to ‘non-specular’ ratio from numerous calculations for the 2θ > 30° region varies from 1.3 × 10^−7^ to 1.7 × 10^−7^, which is in broad agreement with equations (3*a*)[Disp-formula fd3a] and (3*b*)[Disp-formula fd3b]. The increased ‘specular’ contributions captured at low 2θ values is a consequence of the larger capture volume in the axial divergence. The larger capture volume at low 2θ values also increases the number of ‘non-specular’ contributions in a similar way. The consequence of this is that the ‘Bragg’ and ‘non-Bragg’ contributions, given in equation (26), are fairly similar across the 2θ range, as shown in Figs. 13 ▶(*c*), 13 ▶(*e*) and 13 ▶(*f*), for 10 µm perfect crystallites, 3 µm perfect crystallites and 10 µm crystallites with defects, respectively.

It is reasonable to suppose that, for different size crystallites, focus size and Soller slits (the main determining factors) will not change the ratios significantly. It should be remembered that data collected around a complete Debye–Scherrer ring will be composed of intensity with a combination of these ‘specular’ and ‘non-specular’ contributions, and each measurement capture region will follow a very similar ratio.

Two configurations commonly used with the Bragg–Brentano geometry will now be considered in more detail, so that a more realistic estimate of the reliability in the measured intensity values can be assessed.

### 9.4. The case of a stationary sample   

For a sample illuminated with a 10 × 10 mm footprint composed of 10 µm crystallites three deep (half the absorption depth for Si), with a 50% volume density, the scatter will be created from 1 500 000 crystallites when the sample is stationary and held at a specific 2θ. For a reflection at 69° 2θ and a multiplicity of 8 (004 reflection), the average number of Bragg contributions will be 0.5 × 1 500 000 × 8 × (∼1.3 × 10^−7^); the latter is the ratio given above, which can be considered as the likelihood of capturing the ‘Bragg condition’ from a crystallite. This results in ∼0.75 Bragg events and 1.2 × 10^7^ non-Bragg events, for a stationary non-rotating sample (the 0.5× accounts for the scattering going towards −2θ). The σ/*I* values for the Bragg and non-Bragg are ∼88% and ∼0.02% respectively, from equations (26) and (28). The overall σ/*I* is ∼64%. This combination of stable underlying intensity reduces the impact of the very variable ‘Bragg’ or ‘specular’ contributions to give a much more reasonable overall variability.

This overall σ/*I* of ∼64% is the value calculated based on pure monochromatic radiation and a very narrow detector slit. During an actual experiment there is a dispersion of wavelengths and a range of scattering angles entering the detector slit, *i.e.* the limited resolution of the diffractometer helps to reduce the variability. If the radius of the diffractometer is 240 mm, with an X-ray focus of 40 µm, a detector slit of 55 µm for 10 µm crystallites, then from geometry, the spread in 2θ trajectories accepted is 0.041°. The calculated intrinsic scattering width for the 004 reflection from Si is ∼0.0025°; therefore in simplistic terms it can be considered that the effective number of Bragg events could be magnified by 0.041/0.0025 ∼ 16. If the integrated intensity is measured (or the whole shape of the profile is used in the analysis), then the angular acceptance at the detector slit from a 10 µm crystallite at this radius is 0.024°, which is the unique sampling range. For a typical experimental profile width of ∼0.1° this will result in 0.1/0.024 ∼ 4 unique captures over the peak. The accepted wavelength spread (Δλ ∼1.6 × 10^−3^ Å for Cu *K*α_1_ radiation) within these allowable trajectories for each sample point is greater by a factor of 2 than the natural wavelength dispersion of 7 × 10^−4^ Å (FWHM for Cu *K*α_1_), so the effect is small in comparison, adding ∼20% to the width. The inclusion of these experimental aberrations will increase the number of Bragg events to ∼51, and the non-Bragg to 1.6 × 10^9^, which in turn reduces the σ/*I* to ∼14% for the Bragg contributions. However, when the non-Bragg contributions are included, the overall σ/μ ∼8.0%. This estimate assumes that the probability of capturing a Bragg event through scanning is uncorrelated, which may not be the case, and also does not include shot noise *etc*.

### 9.5. The case of a rotating sample   

**Figure 14:**
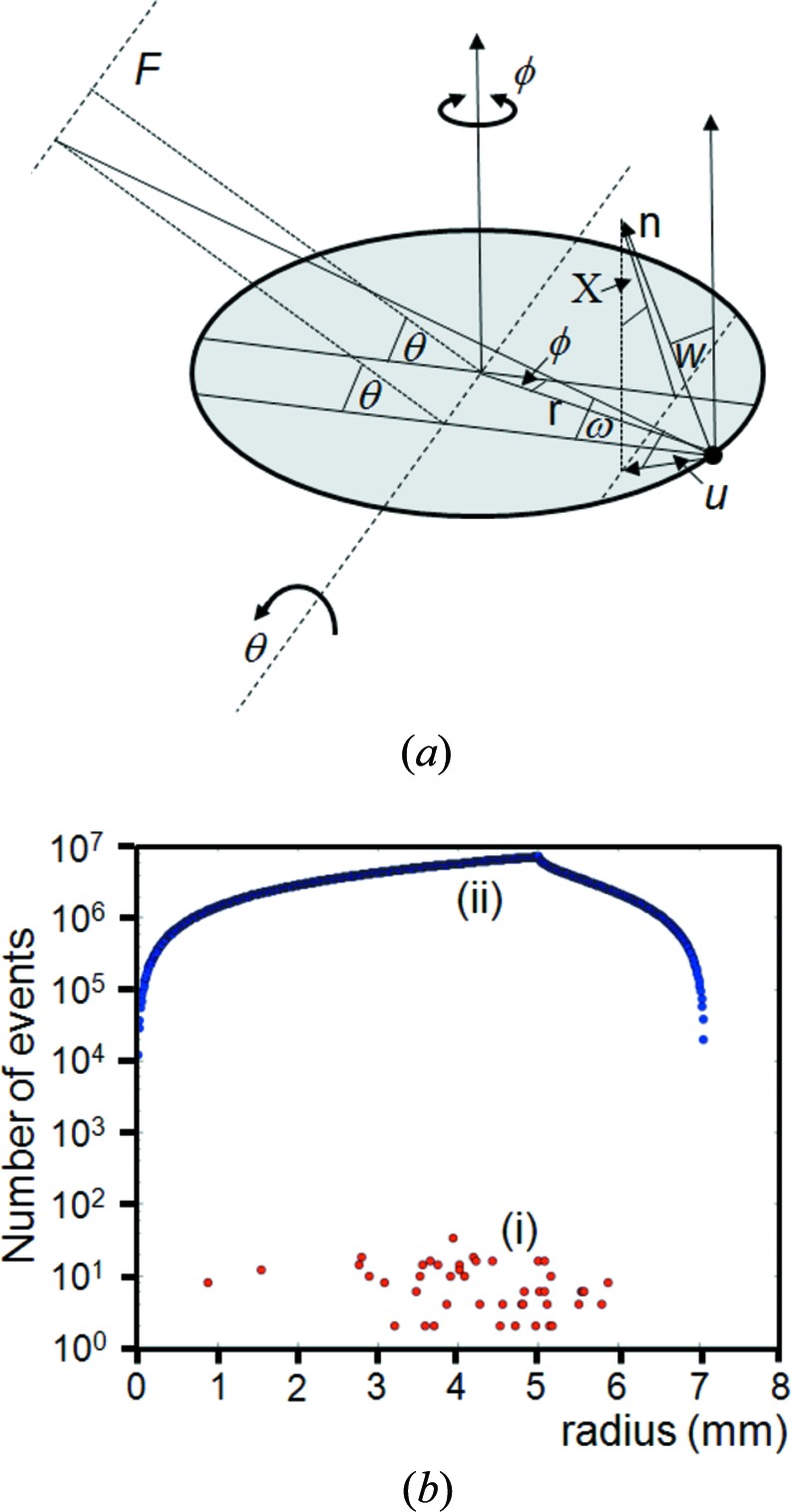
(*a*) The parameters defining the geometry of a crystallite at a distance *r* from the rotation axis ϕ, with orientation coordinates defined by *U* and *W*. (*b*) The total number of ‘specular’ events (i) captured by the detector at 2θ = 60° from various radii as the sample is rotated, and (ii) the number of ‘non-specular’ events that can reach the detector. The sample is assumed to be larger than the illuminated area, which is 10 × 10 mm; the kink in the profile occurs because the illuminated area is square and the fully illuminated area during a full rotation is a circle.

Sample rotation could change the variability and perhaps make the ‘Bragg’ condition more likely. In a typical experiment using the Bragg–Brentano geometry, the sample is rotated about an axis ϕ, normal to its surface. For a crystallite at a distance *r* from the centre of rotation, the incident beam it experiences will vary during rotation. This depends on the angle of its scattering plane normal to the axis of rotation, *W*, and the projection of the scattering plane normal onto the normal of the rotation axis, and perpendicular to the diffractometer θ axis, *U* (Fig. 14 ▶ *a*), such that a crystallite position within the sample can be defined by 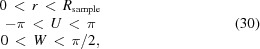
where *R*_sample_ is the radius of the sample. By geometry, the incident angle onto an individual crystallite, *i*, at a distance, *r*, from the centre of rotation at an angle, ϕ, away from the plane including the rotation axis and the diffraction plane is
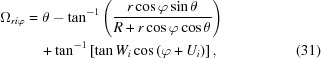
where *R* is the diffractometer radius. The tilt, X, as defined before (Fig. 6 ▶ *a*) can be envisaged as in Fig. 14 ▶(*a*) and is given by 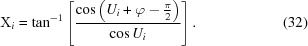
These equations give Ω and X, as in equations (6), (8) and hence (9) as ϕ is rotated for a specific crystallite plane defined by a position *r*, and angular descriptions given by *U* and *W*. A further constraint on the crystallites that contribute is the illuminated length in the diffraction plane, defined by the divergence slits, and the mask that limits the illuminated length normal to the diffraction plane. The calculation is performed by taking a random distribution of crystallite orientations in rings with a dimension of the crystallite diameter, and making very fine steps in ϕ that modify Ω and X for 0 < ϕ < 2π. Clearly, simulating the full pattern from many crystallites during rotation is a very lengthy calculation, so an estimate was obtained by noting when a crystallite is orientated into the specular (and also therefore the Bragg) condition, defined as within the angular acceptance ΔΩ (= 0.017° for 10 µm crystallites) and ΔX, equation (15), and if not when 0 (or π − 2θ) < Ω_X_ < 2θ_B_ (or π/2) for the non-specular (and also therefore the non-Bragg condition). Since the mean specular and non-specular intensity values have already been established for a very large number of crystallites, the number × mean will give the total intensity of the two contributions.

The number of specular and non-specular contributions is given in Fig. 14 ▶(*b*) as a function of the radius, for an illuminated length of 10 mm and a mask of 10 mm. This calculation was repeated ten times to give an idea of the variation, which would also give an indication of the reliability in measuring several samples with randomly orientated crystallites. In one revolution, assuming that only the top three crystallites contribute, which is half the absorption depth in Si, the accumulated number of specular and therefore Bragg events at 2θ = 60° amounts to 378 (108), whereas the number of ‘non-specular’ events amounts to 2 288 677 997 (434 565). A contributing specular event is counted each time a step in ϕ registers one, and therefore a single scattering plane can contribute to more than one specular event. In the example of the contributors in Fig. 14 ▶(*b*) they range from two contributions per specular event up to 32; this indicates that they exist over an angular range in ϕ from ∼0.2° up to ∼3.2°. The ratio of the number of ‘specular’ to ‘non-specular’ events is 1.65 × 10^−7^, *i.e.* within the spread of values mentioned previously. This has been repeated at 30°, and again the ratio is of a similar order, although the average angular range of the contributions per specular event appears smaller. This ratio of ‘specular’ to ‘non-specular’ events is also the same for smaller illumination sizes. Clearly from this analysis the incremental rotation in ϕ can introduce more crystallites into the specular condition, and also allows some to exist over large angular ranges; this also increases the number of non-specular contributions and the ratio is unchanged. The ratio of the intensity contributions is therefore unchanged from the stationary condition, although the number of contributions from these ∼1 200 000 crystallites is increased over that of the stationary experiment, thus reducing the variability.

Undertaking a similar analysis to that above for a 10 × 10 mm footprint and rotating, the σ/*I* for the Bragg contributions is 5.1% and the overall value is 3.2%. Hence a very large sample of randomly orientated crystallites will have reliable results; anything greater than this would suggest preferred orientation, provided shot noise and other effects are accounted for. These values are changed to 0.65% for ‘Bragg’ and 0.40% overall when the aberrations from wavelength dispersion and slits, but not multiplicity, are included. A minor phase at the 10% level in this sample would give an overall σ/*I* closer to 1.2% (or 10.1% without these aberrations) and for a phase proportion of 1%, would give σ/*I* close to 4% (or 32% without aberrations). The equivalent σ/*I* values if they are based purely on ‘Bragg’ contributions would be 2.0% and 6.4% (or 16.3% and 51% without aberrations), respectively. The presence of the ‘non-Bragg’ contributions gives a significant improvement in the reliability of the measured intensities, and similarly for the presence of aberrations. As mentioned above, these aberrations may be correlated and therefore not independent: this will reduce their impact on the σ/*I* values.

It should be remembered at this stage that these calculations of the intensity at the Bragg condition are all based on a kinematical model. The suppression of the peak ‘Bragg’ intensity with respect to the tails from dynamical effects will increase the ‘non-Bragg’ contribution and lower the variability. The above example is for the 004 reflection from Si, which has a multiplicity of 8, and produces a greater number of 004 ‘Bragg’ events per crystallite, compared with a structure of lower symmetry.

### 9.6. Structural aspects that may modify the probability of ‘Bragg’   

During the course of this study, diffuse scattering, crystallite size dispersion and mosaicity were considered as factors that might influence the intensity reliability, the number of Bragg events and the intensity ratios. The diffuse scattering will redistribute the ‘Bragg’ into the ‘non-Bragg’ contribution, whereas size dispersion and mosaicity will introduce more ‘Bragg’ events but reduce their impact. These aspects start to become sample dependent, which could be problematic for an overall general explanation without experimental evidence; however, they are considered and discussed here.

*Crystallite size dispersion*. The effect of crystallite size distribution was tested by calculation of the intensities with different size crystallites and then combining; however, the broad conclusions are the same. As the size is reduced, the intensity dispersed into ‘non-Bragg’ increases and the height of the Bragg peak reduces. In an equivalent calculation as above but for 3 µm crystallites, the ‘non-Bragg’ increases at the expense of the ‘Bragg’ contribution. The proportion of intensity that can be attributed to ‘Bragg’ reduces to 65% compared with 70% for the 10 µm crystallites. These calculations were based on the equivalent of 300 000 000 events at 10° and 2 703 000 000 events at 90° 2θ. The proportion of the crystallites in the Bragg condition is independent of the crystallite size or number; it is the reduction of the intensity in the Bragg condition and the increased intensity in the tails that change the proportions. This is because in general the instrument capture is significantly larger than the intrinsic scattering width of the crystallites; the likelihood of this being the case is considered next.

**Figure 15:**
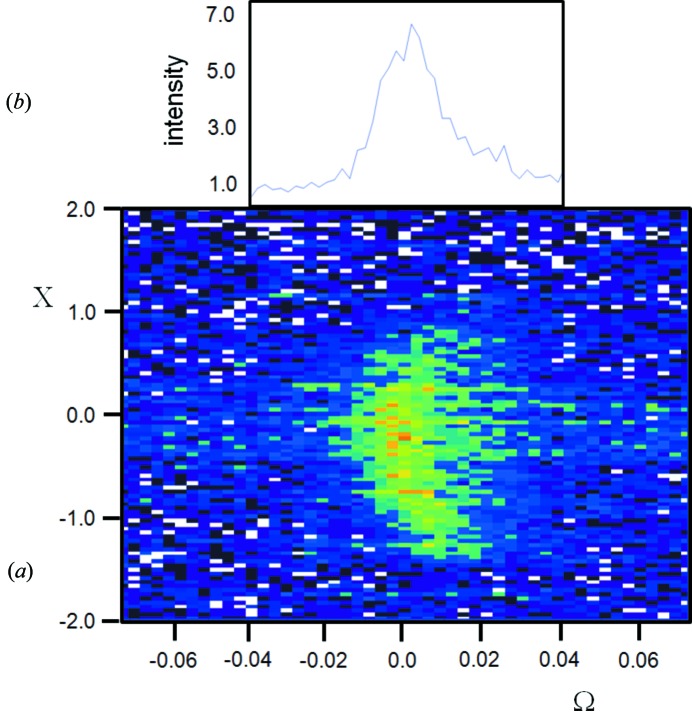
(*a*) The intensity measured in the vicinity of the 220 Bragg peak from a crystallite by isolating it from a larger diffraction space map, measured by Patricia Kidd. The geometry used was the Beam-Selection-Diffractometer (Fewster, 2004 ▶), with a 100 µm double pinhole, Cu *K*α and 220 Ge analyser crystal. The near-perfect match of the reflection choices removes the wavelength dispersion to reveal the intrinsic width of the Bragg peak, which is 0.002°. Along Ω is blurred by diffuse scattering and the pinhole geometry. This can be compared with Fig. 7 ▶, although this extremely high resolution diffraction space map is truncated because of the limited acceptable axial divergence. The maximum intensity is ∼2 counts s^−1^. (*b*) is the projected sum of the central region of the map.

*Mosaicity*. The presence of mosaicity is much more difficult to visualize, since this would require all crystal plane orientations to be dispersed in a similar manner. Yet mosaicity requires the crystallite to be composed of smaller crystallites with similar orientations. These can be joined by low-angle grain boundaries, which are often crystal plane orientation dependent, or if the angles are large, then they scatter independently. This will either lead to some preferred orientation or be equivalent to more small crystallites. By isolating a single crystallite from the Si powder sample under study, the ‘rocking angle’ (scanning in Ω) showed no evidence of mosaic features in the studied sample (Fig. 15 ▶), just a sharp narrow peak ∼0.002° in 2θ (from an accumulation of these diffraction maps) and ∼0.02° in Ω; this is too small to have an effect on the capture of the Bragg condition.

*Diffuse scattering*. Two forms of diffuse scattering are considered to try to understand their influence. These are: thermal diffuse scattering (TDS), see the Appendix[App appa], and more realistically, scattering due to defects, such as dislocations, point defects, clusters and surface damage. The overall intensity with all these additions is unchanged in the kinematical scattering model, but is redistributed in Ω and X, and in 2θ if they introduce a strain. Many authors have studied the defects in Si, including the resulting diffuse scattering, *e.g.* Lal *et al.* (2000 ▶). Lal *et al.* have found that as-grown Si can have oxygen clusters of ∼0.24 µm, which result in diffuse scattering with a characteristic change in intensity fall-off in reciprocal-space distance, *s*, from the reciprocal-lattice point, where this changes from Δ*s* ^−2^ to Δ*s* ^−4^ (Huang, 1947 ▶; Stokes & Wilson, 1944 ▶). Annealing in oxygen above 873 K reduces the width of the diffraction profile. The concentration of defects in as-grown Si gives observable diffuse scattering at the 10^−1^ *I*_0_ level, above that expected from TDS. In reality, surface damage could increase diffuse scattering significantly (Fewster & Andrew, 1993*b* ▶). If diffuse scattering is observed at the level modelled here in semiconductor-grade Si, then it is likely to be present in virtually all samples.

Typical preparation methods will almost certainly produce significant levels of surface damage and hence diffuse scattering. Knowing whether this is a true representation of the diffuse scattering is not essential, but knowing whether it can influence the intensity ratios is important. The analysis similar to that above gave results very similar to those in Figs. 13 ▶(*a*) and 13 ▶(*b*), indicating that at the 3 × 10^−1^ *I*_0_ level of diffuse scattering, the expected intensities are indeed unchanged in the kinematical model, *i.e.* the significant part of the diffuse scattering is generally within the same capture volume as the Bragg peaks. The proportion of ‘specular’ to ‘non-specular’ was closer to 75%, with a standard deviation of 6%, although as the diffuse scattering increases this will reduce this value (Fig. 13 ▶ *f*). The defects giving rise to the diffuse scattering can, though, change the dynamical/kinematical ratio.

**Figure 16:**
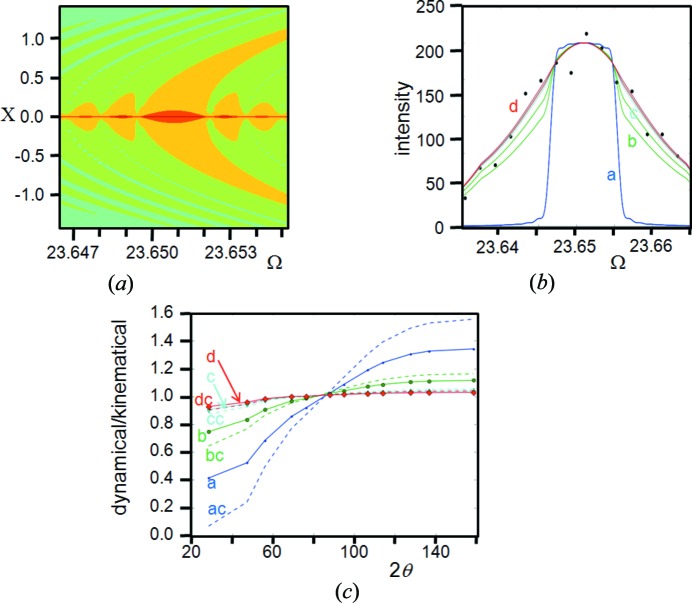
(*a*) The simulated scattering within the instrument capture volume for 10 µm crystallites at the exact Bragg condition within the Bragg–Brentano geometry (Si 220 reflection) with no instrumental aberrations or diffuse scattering. (*b*) The simulated profile (including instrumental aberrations from the geometry given in the caption to Fig. 15 ▶) with increasing levels of diffuse scattering, a, b, c, d, that correspond to the ratios of the peak in the diffuse scatter to the kinematical maximum of 0, 0.1, 0.5 and 0.75, respectively. The barely observable double line for each corresponds to transmission (lower line) and reflection (upper line) dynamical contributions. (*c*) The residual dynamical contributions for the Bragg–Brentano geometry described in the text; the intensities are given for the geometric average of the reflection and transmission geometries, with the dashed line for conventional theory (ac, bc, cc, dc) and the solid line for the new theory (a, b, c, d), where the fractions correspond to those in (*b*).

If it is considered that the minimum diffuse scattering estimated by Lal *et al.* (2000 ▶) is ∼10% of the 111 Bragg peak intensity, then this gives a starting value for the level of diffuse scattering to include. The ratio of the intensities for 10 µm spherical crystallites based on the dynamical and kinematical theories is ∼0.08 (using the geometrical mean of mixed contributions as described above: in pure reflection and transmission this would be 0.12 and 0.04, respectively). These evaluations are calculated by including the full capture volume (Fig. 16 ▶ *a*), with the addition of the influence of the detector acceptance, Δ2θ_s_. If diffuse scattering is included at 10% of the kinematical peak level, then the ratio tends towards ∼0.63 (0.78 and 0.49 in reflection and transmission, respectively).

It is clear that the impact of diffuse scattering could be significant in reducing the influence of dynamical scattering on the intensities and may explain the near-perfect fit to the kinematical theory. The data presented in Fig. 16 ▶ serve as a useful guide as to how much diffuse scattering should be included, based on the 220 reflection. The simulation of the profile in Fig. 16 ▶(*b*) is obtained by first calculating the Ω profile, assuming kinematical scattering for a perfect crystallite, and including diffuse scattering with a magnitude defined by a ratio of its maximum to the original peak intensity; the total intensity is assumed to be the same. The remainder of the kinematical proportion that is not associated with the diffuse scattering is then subjected to dynamical scattering to give an approximation of the scattering from the perfect and imperfect regions.

The profile to be compared with the experimental result includes the instrumental aberrations (Fig. 16 ▶ *b*): the crystal analyser has an acceptance ∼0.003° and is set to the Bragg peak, and the angular acceptance by the crystallite is given by the crystallite–focus combination, which gives the blurring of the profile in Ω, and is of the order of 0.008°. The experiment used a double pinhole in combination with a line focus and a three-bounce 220 Ge analyser crystal to remove wavelength dispersion (Fewster, 2004 ▶). The level of diffuse scattering was varied until the profiles became roughly coincident (Fig. 16 ▶ *b*). The experimental profile, shown as dots in Fig. 16 ▶(*b*), has a slower fall-off in intensity than that predicted using a defect size of 0.24 µm (b and c in Fig. 16 ▶ *b*). The best fit occurred with a defect size of ∼0.18 µm (d in Fig. 16 ▶ *b*) with a diffuse peak to kinematical peak fraction of ∼0.5. However, it is not the purpose of this article to analyse the diffuse scattering in detail, but to indicate the level of dynamical scattering that is likely to be present, by introducing diffuse scattering to account for the measured profile. The diffuse scattering is assumed to be kinematical.

This estimate of the diffuse scattering contribution is then applied to all reflections, with the capture volume for the Bragg–Brentano geometry used in this study. This gives the ratio of the dynamical/kinematical theories, from which the scale factors can be determined to correct the intensity contributions for dynamical effects when the Bragg peaks are captured (Fig. 16 ▶ *c*). From Fig. 16 ▶(*c*) it appears that the level of diffuse scattering is in excess of that required to modify the kinematical assumption by more than a few per cent. It is also important to note that the profile of the diffuse scattering does not change significantly as the fraction is increased above 0.5, so this is really a lower limit to account for the experimental results, and therefore the dynamical effects are likely to be less than this. This methodology also assumes that there is a clearly defined perfect and imperfect region, which is unrealistic but does represent the maximum impact that dynamical effects can have. It seems reasonable to assume that as the defects become more distributed then the perfect region size shrinks and the dynamical effects are further diminished.

It is worth noting that dynamical effects will have a bigger impact on conventional theory than on this new theory for powder diffraction. The intensity is assumed to only come from Bragg scattering in the former, whereas this new theory reduces the proportion of intensity from Bragg scattering to ∼0.75 for 10 µm Si crystallites. As discussed earlier, this is the limiting case, and for many experiments, when there are far fewer crystallites, the variability of intensity associated with Bragg events will mask the dynamical effects. For the sample composed of 2.3 × 10^9^ possible orientations given in Fig. 14 ▶, the number of Bragg events is 380 with a variability of ∼10% which is comparable to the influence of dynamical effects, and therefore the impact of dynamical theory will be masked. Also, if the diffuse scattering is significant then it will redistribute the scattering outside the Bragg peak capture volume, raising the ‘non-Bragg’ contribution, which in turn reduces the ‘Bragg’ peak intensity and therefore the proportion of Bragg scattering. If the impact, of encountering a Bragg peak, on the measured intensity becomes less significant, then the data may be less variable, which is the case when smaller crystals are used. For smaller crystals it would be reasonable to assume that the ‘defect to perfect’ crystal ratio increases with the ‘surface roughness to volume’ ratio. It appears that kinematical scattering is a good approximation to the scattering observed from crystals with defects, *i.e.* ‘real’ crystals; however, we can draw some other conclusions that are covered in the following section.

## 10. Comparisons between theory and experiments   

Equation (17) represents the intensity arriving at the detection point 2θ, and each scattering plane will have contributions to each 2θ value that fit within the bounds. The full profile can therefore be calculated at each value of 2θ from 0° to 180° (or 4θ_B_ if smaller). The emphasis in this section is on Si, since the atom positions are defined by symmetry and the temperature factors have been measured and calculated to high precision (see the Appendix[App appa]). The comparison in intensity will be with the Bragg–Brentano geometry, because this is the most widely used configuration. An explanation for the intensity features observed in a Debye–Scherrer ring will also be illustrated.

### 10.1. Bragg–Brentano geometry   

The basic Bragg–Brentano configuration used in this study consisted of a goniometer of radius 320 mm, an incident beam slit of 0.125°, a projected focus size of 0.0418 mm that results in an illuminated area of ∼0.7/sin θ mm (giving a maximum and minimum area of 2.8 to 0.7 × 12 mm over the range of reflections possible for Cu *K*α radiation) and this ensures that the illuminated area is still smaller than the sample at 10° 2*θ*. This configuration should ensure that exactly the same total intensity is experienced at each angular range, although the number of crystallites will vary. The detector used was a solid-state PANalytical PIXcel, with data collected in 0.055 mm strips, producing good para-focusing conditions. Soller slits of 0.04 radian were placed in the incident and scattered beam to limit the cross-fire. The sample was a disc composed of an amorphous resin and Si crystallites in equal proportions. The Si crystallites have dimensions ranging from 5 to 15 µm, with > 80% being 10 µm.

**Figure 17:**
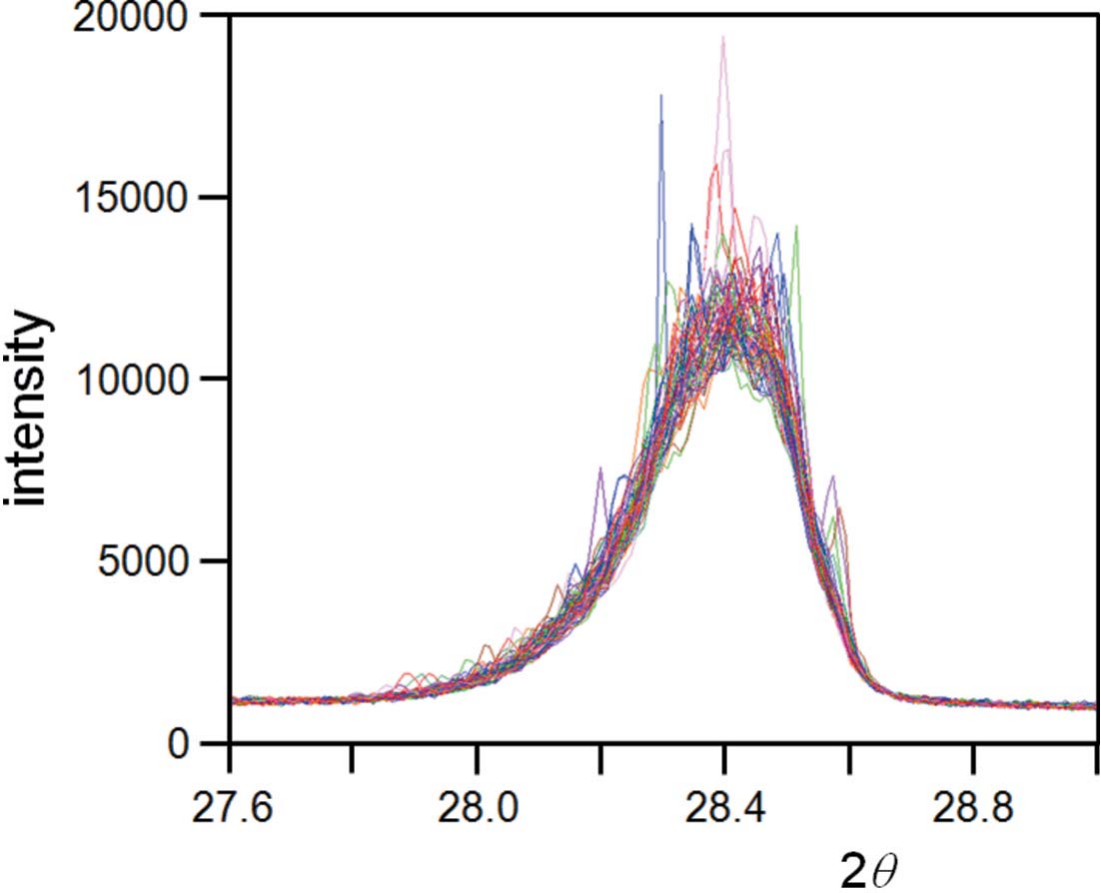
A series of stationary profiles, using the Bragg–Brentano geometry, of the Si sample described in the text. The experiments were measured at 0.5° intervals over 10° in ϕ. The profiles appear to exist in two distinct forms, a general ‘background’ (smooth hump) and a spiky region superimposed, thought to be associated with ‘non-Bragg’ and ‘Bragg’ contributions, respectively. The spikes appear and disappear within similar angular ranges in ϕ as predicted from the calculations leading to Fig. 14 ▶(*b*).

For a 30 µm penetration these dimensions equate to ∼26 000 crystallites at 28.4° 2θ and 6300 crystallites at 158.6°, illuminated at any one time at each data-collection point. This would equate to ∼0.25 × *M_hkl_*/2 (∼1 for *hkl* = 111; *M_hkl_*/2 takes account of the distribution of scattering towards 2θ and −2θ) and ∼0.05 × *M_hkl_* (∼0.25 for *hkl* = 444) Bragg events at each of these two angles, when all the factors of wavelength dispersion and divergence are included as discussed previously. The non-Bragg events are similarly increased to 6 × 10^6^. The estimated σ/*I*, for six independent measurements (experimental details are contained in the following paragraph), varies from ∼10% to ∼30% (Fig. 2 ▶), and is comparable to the calculated σ/*I* for the condition with no aberrations of ∼22% and greater than the condition with aberrations of ∼2.7%. The latter value is the estimated best result that could be achieved, remembering that this does not include shot noise, and assumes that the contribution from scanning is uncorrelated: if it is correlated this latter value may increase up to a limit of ∼11%. If the number of independent measurements was increased tenfold then the σ/*I* value would be within the bounds of 3% to 10%, so these calculated and measured σ/*I* values are in broad agreement. These are only estimates, so to support this, a series of stationary measurements of the 111 reflection were taken at various ϕ angles (Fig. 17 ▶). The divergence slit was set to 0.0625°, 0.04 radian Soller slits and the data were captured on the PIXcel as a one-dimensional strip detector, which is why the resolution is poorer than in the scanning method. The profile can be seen to consist of two parts, a spiky part superimposed on a well defined hump: this can be interpreted as the Bragg spikes superimposed on top of non-Bragg contributions. The number of significant peaks per scan is ≤1, which fits with estimates above for the 111 reflection. It is difficult to judge exactly where the Bragg to non-Bragg boundary should be, but from visual inspection it appears that the non-Bragg intensity is greater than the Bragg intensity in this experiment (Fig. 17 ▶), whereas in the limit of very large numbers of crystallites that are perfect, the Bragg intensity is greater than the non-Bragg intensity (Fig. 13 ▶ *c*). It is clear though that the intensity arriving on each strip has a stable non-Bragg intensity contribution with a much more variable Bragg intensity contribution superimposed.

**Figure 18:**
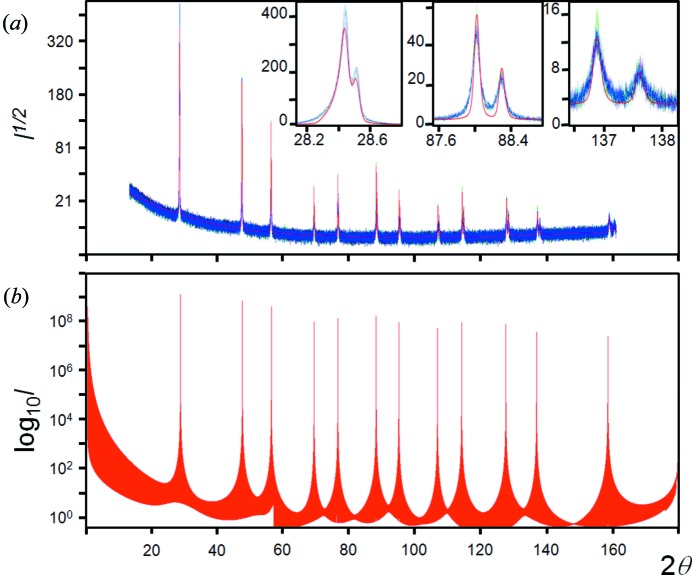
The full profile for Si powder with Cu *K*α radiation with (*a*) and without (*b*) the instrumental artifacts; (*a*) and (*b*) are plotted on square-root and logarithmic scales, respectively. The calculated profile is given by the thick red smooth line, and the six experimental profiles in (*a*) are overlaid to give some indication of the variations between samples, which is covered in more detail in Fig. 19 ▶. The artifacts included are all defined by the instrument dimensions, except the background due to residual scattering (presumably hard radiation from the slits *etc.*), which is fitted with a polynomial. The spectral dispersion, *e.g.* Cu *K*β after absorption by the Ni filter, appears at the right level within the background noise. A few reflections are plotted on a linear scale to illustrate the agreement.

To determine the intensity ratios between all reflections, a series of 2θ/ω scans in the centre of six different samples from 10° to 162° were collected whilst spinning the sample in ϕ. These gave an average value and a variance for the integrated intensity. The count time was 8.67 s per 0.0025° step captured in continuous collection mode (Fig. 18 ▶ *a*). The integrated intensity of a reflection in a typical diffraction experiment is given by the summation over 2θ close to the peak. The region of integration will be limited by the dynamic range of the experiment and the general shape of the 2θ profile, but as discussed earlier this will only represent a proportion of the scattering power. Three representative measured peaks are given as insets to Fig. 18 ▶(*a*), and from these it is possible to estimate the proportion of the scattering power captured. The integrated intensity has been taken as the sum of the contributions above the ‘background’. The location for choosing the background level for the experiment indicates the angular range for estimating the equivalent integrated ‘peak’ intensity in the calculated profile. The proportion of the scattering power that is captured in the experiment for the various 2θ values is given in Fig. 11 ▶: it is also important to remember that it is also dispersed in 2θ. By measuring the intensity in a systematic way, it should be possible to obtain a good estimate of the proportion of the dispersed scattering power.

This theory creates the full profile as discussed before; however, to bring it into line with the actual experiment, there are some additional factors to consider, some of which have been alluded to in estimating the σ/μ.

(*a*) Wavelength dispersion: in effect, each wavelength constitutes a different experiment, so a convolution is a reasonable approximation, since the whole simulation can be repeated for each wavelength increment and the conclusions would be unchanged apart from the 2θ cut-off bound. In this article the width Δλ is derived from uncertainty in the energy-level transition in a Cu atom and amounts to ∼0.0007 Å, and both *K*α_1_ and *K*α_2_ are assumed to be present. The shape should include the whole wavelength distribution. An Ni filter was used without a monochromator, and since there is no simple representation of the tube spectrum and detector response, this was measured with a perfect Si 111 orientated wafer. The wafer was scanned from 8° to 50° in 2θ, coupled to ω, and converted to a wavelength scale to create the wavelength dispersion convolute. The measured width of the *K*α_1_ peak equated to 0.00098 Å, close to the theoretical estimate, from a width of 0.02° 2θ, which is large compared with the monochromatic theoretical width of 0.004° based on dynamical theory, and considerably less than this based on kinematical theory. To match the theoretical *K*α_1_ peak width of 0.0007 Å, the profile was fitted to a Cauchy-squared profile, then modelled with the FWHM × (0.0007/0.00098). The difference between the two calculated profiles was then added to the measured profile to create the theoretical width, whilst keeping all the artifacts associated with the filter, detector response and broader spectral emission unchanged.

(*b*) The detector slit will obviously capture over a range of 2θ values defined by Δ(2θ)_s_. 2θ will vary depending on the origin of the photon emission from the focus and the crystallite size (*i.e.* the spread in the scattering centre); these three components result in a simple geometric relationship of possible paths, and by taking into account the focus emission, a further Gaussian convolute is a good approximation.

(*c*) The data were collected with a solid-state strip detector (making full use of all 255 strips), meaning that the sample orientation is only optimized to the central strip whilst scanning. Therefore a small smearing will occur in 2θ, depending on the angular range of the detector; again this is another simple geometrical relationship. In this case, the convolute is rectangular in shape as it happens to be a very small effect.

(*d*) Sample scattering depth: this can be calculated from the photoelectric absorption, to estimate the intensity contribution as a function of depth, which is again a convolute and results in a small displacement bias in the actual 2θ scattering angle, that is a function of 2θ.

(*e*) Axial divergence effects: these result in a redistribution of the intensity along 2θ because not all scattering lies in the plane of the diffractometer. The result is a projection effect that is most pronounced at high and low 2θ values. If this angular displacement is δX then the measured intensity will appear at 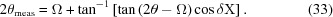
This can be included as a convolute whose shape was first given by van Laar & Yelon (1984 ▶), with minor corrections given by Finger *et al.* (1994 ▶). These works assume that there are a large number of crystallites, which will be relevant to the example given here.

These geometrical influences on the peak shapes assume that the instrument is well defined and perfect, when in reality this may not be the case, *e.g.* alignment of the focus with the sample surface and with the detector strip. Other influences on the profile could include: a distribution of crystallite sizes, strain variations between crystallites or strain variations within a crystallite. However, if we compare the integrated intensities, then all these effects that redistribute the intensity are captured, and a true comparison can be made between theory and experiment.

The calculation of the full profile over the angular range is given in Fig. 18 ▶(*b*) for the case with no instrumental aberrations, whereas the experimental profile is overlaid with the calculated profile (including aberrations) in Fig. 18 ▶(*a*). The calculation does not involve fitting, apart from scaling, and introducing a background that is largely dominated by the scatter from the slits *etc.*; the hard radiation component is likely to cause fluorescence. The insets indicate the peak comparisons, assuming every crystallite is unstrained and all are the same size; the small differences can be simply analysed (but not presented here) by adding some strains and size distribution that could explain the residual broadening effects. Since each crystallite orientation will contribute to every ΩX map at every 2θ, provided that the X-ray beam impinges and scatters above the plane and on opposite sides of the plane normal, the number of sampled regions within the ΩX map is very large even for only a few crystallites. The ‘background’ observed in 2θ is the sum of all the contributions from all the reflections (Fig. 18 ▶ *b*).

**Figure 19:**
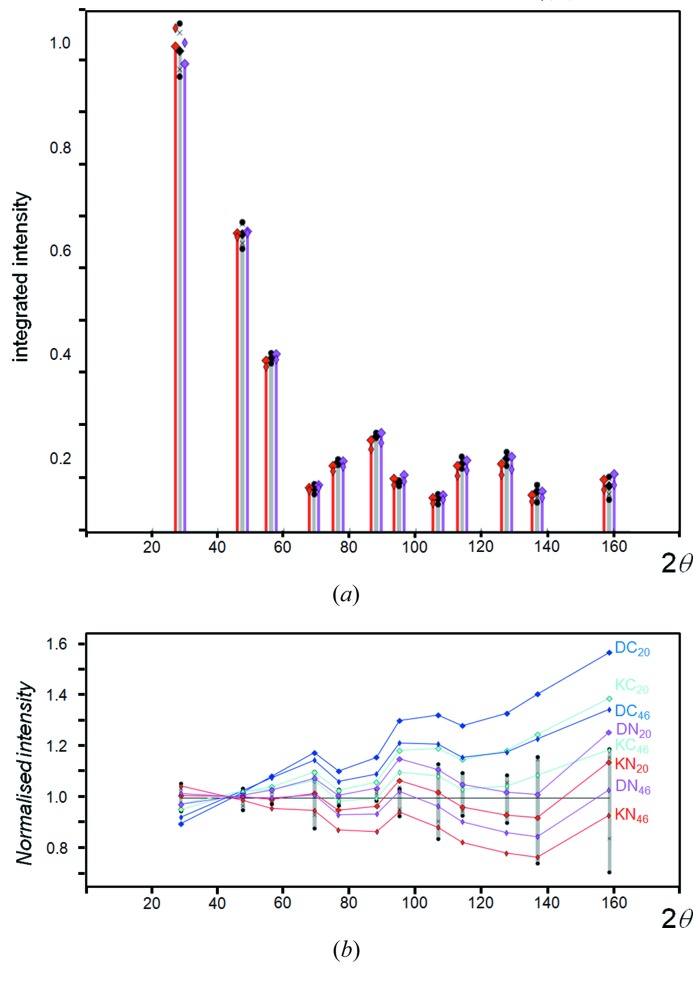
(*a*) The integrated intensity for the measured reflections (grey central bars) displayed as a bar graph for an Si sample (Si crystallites immersed in a resin that occupy 50% of the volume), compared with the new theory (magenta right-hand bars, including dynamical effects, large diamonds for *B* = 0.02 Å^−2^, small diamonds for *B* = 0.046 Å^−2^). The red left-hand bars give the intensities based purely on the kinematical model. The experimental data were captured as described in the caption to Fig. 2 ▶. (*b*) includes the data illustrated in Fig. 2 ▶ and Fig. 19 ▶(*a*), but normalized to the mean intensity to illustrate the systematic trends in the intensities from the new and conventional models. The same nomenclature and colours are used as in Figs. 2 ▶ and 19 ▶(*a*).

The comparison of the integrated intensities is given in Fig. 19 ▶(*a*) and as normalized intensities in Fig. 19 ▶(*b*). The angular range for obtaining the integrated intensity was the same for the experimental and calculated profiles, with the range defined when the experimental profile is indistinguishable from the background. The agreement of the calculated to measured intensities is very close, and a significant improvement compared to the conventional theory (Fig. 2 ▶). The plots include both kinematical and dynamical models and the most likely *B* factor 0.02 nm^2^ and the maximum possible *B* factor 0.046 nm^2^ for Si. The conventional theory always shows a systematic trend away from the experimental intensities for any combination, with the closest fit when *B* = 0.046 nm^2^ and kinematical theory. A *B* factor of 0.06 nm^2^ and kinematical theory is required to bring the trend line into agreement, which is unrealistic. The new theory gives good agreement with the most likely *B* factor of 0.02 nm^2^ and with dynamical effects; also the measured intensities are easily accommodated within the bounds defined by kinematical and dynamical theories and the acceptable range of *B* factors.

### 10.2. Pinhole and area detector   

For completeness, another simulation has been performed for an instrument consisting of a pinhole (small crystallite numbers) and a two-dimensional detector. The summation should be conducted over each pixel, and in this case, it is the Debye–Scherrer ring that is observed (Fig. 10 ▶), which lies along χ. The intensity contribution at an angle χ for a given crystallite is derived from a lattice plane tilt of X, given by
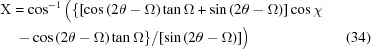
and the incident angle, Ω, and through equation (9). The estimate of the intensity from any of these data-collection procedures is therefore a summation of the three-dimensional distribution of amplitudes, extending in X, or χ, Ω and 2θ. The bounds in χ are unrestricted, *i.e.* 0 to 2π, whereas 2θ ranges from 0 to π. Each crystallite plane will contribute intensity to half of the ring, much of which is weak, although spots will appear when the Debye–Scherrer ring intersects a significant tail (Fig. 7 ▶). The total intensity from a powder sample is calculated by summing all the contributions from all the crystallites at each position on the ring.

**Figure 20:**
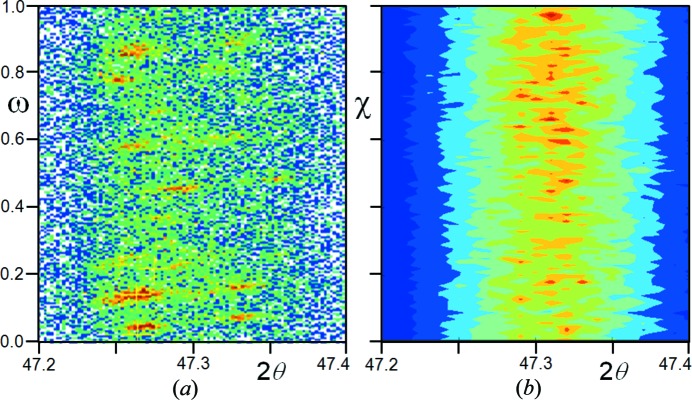
(*a*) The distribution of intensity around the Debye–Scherrer ring (Si 220 Cu *K*α_1_) obtained with a double pinhole (divergence ∼0.1°) and a two-dimensional detector (PIXcel) by scanning in ω and 2θ coupled together, *versus* ω. (*b*) The calculated intensity distribution from 10 000 randomly orientated crystallites captured in 2θ and offsetting in χ. Both geometries are alternative ways of investigating the Debye–Scherrer ring, the former was to achieve controllable high-resolution data. Both images are plotted on a linear scale and follow similar distributions.

The geometry discussed here is the simplest experimental conceptual configuration, with a narrow pencil beam with a divergence of typically 0.1° impinging on a cluster of crystallites. The forward scattering is then captured with an area detector, and of particular interest here is to examine the intensity distribution in the Debye–Scherrer ring. Experimentally this distribution is quite uneven (Fewster & Andrew, 1993*a* ▶, 1999 ▶) and given in a more recent experiment (Fig. 20 ▶ *a*). The conventional interpretation is that the weaker intensity is associated with small crystallites and the more intense spots with larger crystallites that scatter more strongly (see, for example, Brindley, 1945 ▶; Gonzalez, 1987 ▶). The simulation in Fig. 20 ▶(*b*) is constructed from 10 000 randomly orientated 10 µm spherical Si crystallites. The distribution is clearly uneven and mimics that observed in the experiment in Fig. 20 ▶(*a*). It also resembles the variability observed in Fig. 17 ▶, *i.e.* a largely invariant hump of intensity with high points superimposed. Clearly this new theory accounts for all the experimental data, in terms of intensity magnitude and distribution and the profile shape, without recourse to a distribution of sizes, mosaicity, additional aberrations associated with the instrument *etc.*

## 11. The relationship between the new and conventional theories   

The most significant difference between the new theory and conventional theory is that the scattering in the former exists almost everywhere, whereas in the latter, the only scattered intensity is in the immediate vicinity of the Bragg condition (Bragg, 1925 ▶). This explanation is applicable to scattering from single crystals as well as powder diffraction, but the consequences will have a different emphasis in both types of analyses, so it is appropriate to categorize the effects.

### 11.1. Intensities in powder diffraction   

The profiles in conventional theory are typically constructed as a stick pattern with a magnitude defined by the intensity given in equation (1), convoluted with a pseudo-Voigt, Pearson VII or similar function whose width is given by Caglioti *et al.* (1958 ▶) (see, for example, Young, 1993 ▶). These parameters are adjusted to match the measured data, using the method of Rietveld (1967 ▶, 1969 ▶), where the full profile can be simulated. This does assume that the parameters are well defined and the correlations are accounted for in the resulting values.

In this new theory the profile is given by equation (11) by calculating the intensity associated with the capture volume at the appropriate 2θ values through the whole profile. Although the calculation, at this stage of ‘computer code maturity’, is a slow process in comparison to the conventional approach, it does give an indication of the reliability of the measured data depending on the number of crystallites, the impact of dynamical theory *etc*. Thus the whole scattering pattern can be calculated including the peak shapes and the ‘background’, based on the crystallite dimensions. If the whole geometry of the instrument is included, for example slits in the incident and scattered beam and wavelength dispersion, then the whole profile can be modelled without adjustable parameters apart from the residual scattering from the slits (probably from the harder X-rays). This assumes that the content of the sample is known: in this study the amorphous resin binder has been ignored.

This new theory explains why a scattering pattern can be observed with very few crystallites (even just one crystallite can give the full array of peaks; Fig. 8 ▶), by simultaneous scatter from several crystal planes, almost irrespective of their orientations. The individual intensity contributions may be very weak, and some of this scatter will be inaccessible because of the diffractometer geometry. However, the large number of these contributions produces a significant intensity comparable to that of Bragg scattering, and is the reason for the remarkably stable intensities from a limited number of crystallites in the sample.

Some of the experimental data, such as spottiness on Debye–Scherrer rings, can also be simply explained as crystallites orientated close to the Bragg condition. The general hazy background corresponds to the expected scattering based on this theory, whereas in the conventional theory the explanation assumes large crystallites in a mass of small crystallites.

**Figure 21:**
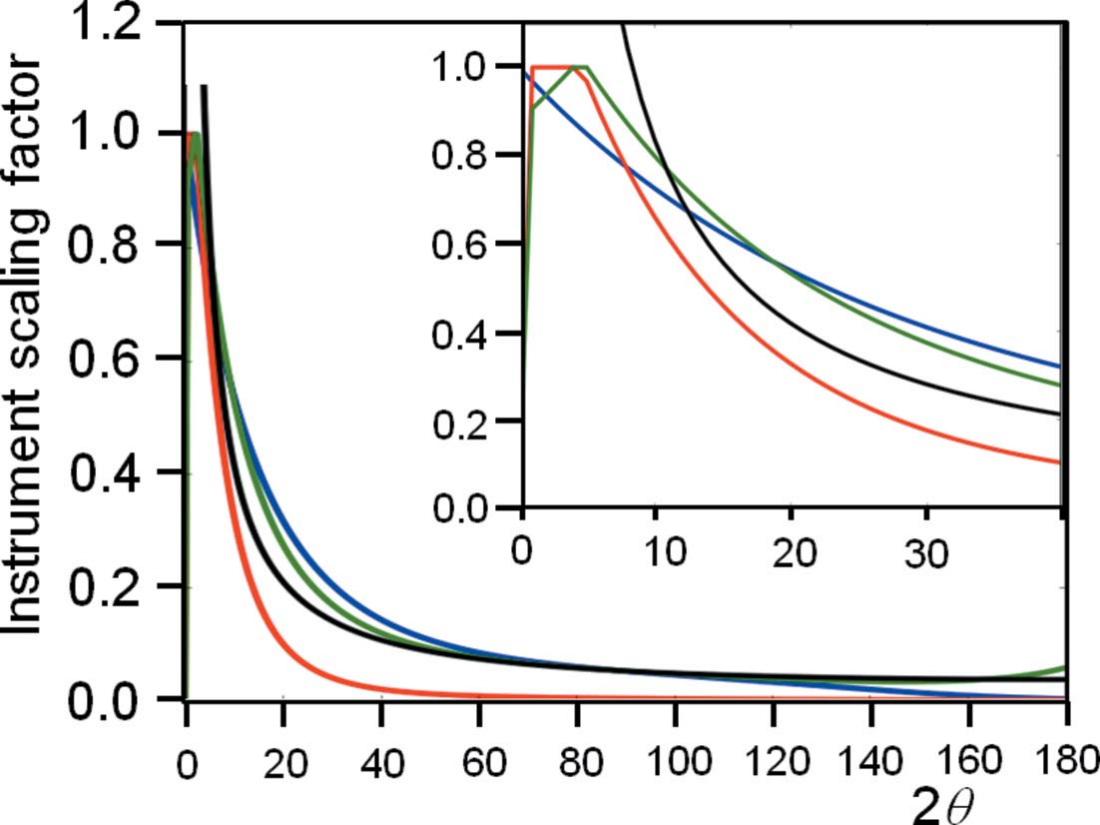
The normalized trend lines for the integral of the dispersed intensities, taking into account the oversampling (red), the integral without oversampling (blue) as in Fig. 11 ▶, and their ratio (green). These are compared with the 1/sin θ (black) geometrical factor used in the conventional theory.

The conventional theory introduces the Lorentz factor and a geometrical factor, equation (1). In the new theory, the former is simply related to the capture volume in diffraction space, equation (15), rather than the time for a peak to pass through the Bragg condition. This capture volume is therefore much more general and applicable everywhere. The geometrical factor 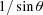 is less obvious in the new theory, since the oversampling falls off faster than this factor. The ratio of the oversampling to the intensity dispersion in ΩX follows this factor approximately, but deviates at angles 2θ < 45°. This suggests that the conventional theory may underestimate the calculated intensities in this range, which is observed in Fig. 2 ▶. For 2θ < 12° the conventional theory will overestimate the intensities that progressively diverge towards 2θ = 0 (Fig. 21 ▶). In the new theory there is a natural cut-off in intensity at twice the critical angle, but prior to that, the range in X has been captured, the full accessible axial divergence has been captured, and the intensity remains constant, which is a function of the axial divergence accepted by the Soller slits or detector.

The intensity is heavily dispersed outside the Bragg condition in both ΩX and in 2θ and, therefore, so is the scattering power. The conventional theory does not take this into account, but associates the scattering power only within the immediate vicinity of the Bragg condition, leading to an overestimate of the scattering power captured, which varies as a function of 2θ. This leads to overestimated temperature factors when fitted to experimental data. Both theories depend on the impact of dynamical effects, which is larger for the conventional theory, and it seems likely that defects play a role in reducing this impact through disruption of the X-ray wavefields and the creation of diffuse scatter.

The equations associated with the new theory allow the intensity from a single crystal, or a collection of crystallites, to be calculated for any position in diffraction space, given the orientation angles ΩX for the crystal planes.

### 11.2. Intensities from single crystals   

The aspects concerning the Lorentz factor are similar to those described above, *i.e.* an oversampling in reciprocal space, rather than the time for the Bragg peak to pass through the diffraction condition. The Lorentz-factor equation is unchanged, but this new description shows that it is valid throughout diffraction space, which is relevant to interpreting the diffuse scattering *etc*.

In the determination of molecular structures, it is assumed that the crystals under investigation are ‘ideally imperfect’; that is they are composed of sufficiently small mosaic blocks that dynamical theory does not need to be invoked. This also leads to the rather arbitrary choice of whether to remove reflections or reduce their effect with ‘extinction corrections’ if the model structure does not match the intensity. Typical crystals are large, ∼500 µm, and dynamical theory would be expected to be very relevant for determining the intensities of intense reflections, since significant dynamical effects can occur at the micron level. However, it can be seen that crystal defects can rapidly diminish dynamical effects; this could be checked quite easily by performing a high-resolution scan to check the validity of using kinematical theory (Fig. 16 ▶ *b*). This could lead to better estimates of the intensities. The complication associated with dynamical effects is that the transform from intensity to scattering power is less transparent, although this prior knowledge could assist in structure determination.

In the single-crystal experiment, the intensity is only captured in the vicinity of the Bragg condition. For an integration volume within 0.01° in Ω and 0.1° in X of the Bragg condition, the intensity from a single 500 µm crystal, compared to the total scattering, is ∼95% assuming kinematical scattering. So, for a larger integration volume defined by ΔΩ ∼ 0.344°, ΔX ∼ 0.344/sin θ, Δ2θ_s_ > 0.38° that is probably more typical (based on an X-ray point focus of 0.4 mm, with 0.5 mm diameter crystal at 150 mm away and a 0.17 mm pixel size detector at 100 mm from the crystal and the intensity is captured within one pixel), the error in associating the measured intensity with the scattering power is not significant. It may be more appropriate to consider an ‘ideally imperfect’ single crystal used for molecular structure determination to consist of defects giving rise to localized diffuse scattering.

Although the crystals are larger than those used in powder studies, these tails are weaker with respect to the Bragg peak and therefore more difficult to observe. However, from equation (9) the absolute magnitude is comparable since the tail amplitudes are increased according to the number of contributing scattering planes, *N*, if dynamical scattering effects are ignored. Thus weak ‘powder’ rings may be observed.

## 12. Conclusions   

This new theory suggests that the observed diffraction peaks from a crystal or crystallite are not composed entirely of those satisfying the ‘Bragg condition’. This has particular relevance to polycrystalline samples, where each crystallite can contribute to many diffraction peaks simultaneously, resulting in the whole diffraction profile building from a limited number of crystallites. This explains the good reliability for the measured intensities, despite the low probability of capturing the Bragg condition. One of the examples given here for perfect 10 µm crystallites of Si (which probably represents highly perfect material compared with typical polycrystalline aggregates) shows that the intensity contribution from crystallites in the Bragg condition may only represent ∼70% of the total scattering, and for smaller crystallites this proportion falls. If additional contributions, such as surface damage from preparation, were included, then the influence of the Bragg scattering would be weaker still, and also reduce the impact of dynamical effects. The consequence of this description is that the intensities differ from the conventional model in subtle ways, which improves the agreement between the calculated and measured values. These differences indicate that the temperature factors may be overestimated, and the low 2θ angle reflections could be overestimated below 12° and underestimated between 12° and 40° in the conventional theory.

The conventional theory in general assumes the crystals or crystallites are ‘ideally imperfect’, which is used to justify the ignoring of dynamical effects. The analysis of the scattering from a single crystallite indicates that there is measurable diffuse scattering, and it is this that explains the suppression of the dynamical effects. The proportion of diffuse scattering required to suppress dynamical effects is less in the new theory compared with the conventional theory for powder diffraction experiments. The extent of the diffuse scattering can be measured, thus indicating the strength of dynamical effects, and could well be useful for the determination of structures from single crystals.

Because the scattering is more ubiquitous than in the Bragg concept, the scattering power (structure factor) from any given reflection is also distributed, and most experiments will not capture all the associated intensity for comparison with calculated models. This becomes more evident with very small crystals. The distributed scattering also accounts for the general hazy band of scattering around a Debye–Scherrer ring, which is often punctuated with tail intersections or near-Bragg events of higher intensity. This new theory can also be used to determine the scattering at any position in diffraction space, whatever the orientation of a crystal.

This description of X-ray scattering has wider implications than just for powder diffraction. Take, for example, some of the latest work on X-ray free-electron lasers, where microcrystallites are streamed to intersect the pulsed beam to produce scattering patterns. Conventional thinking suggests that the number of crystallites required to build up a pattern would be considerable, and subject to questions of reliability, whereas this new theory indicates that the reliability may be quite high without the need for vast numbers of crystallites.

The most obvious conclusion relates to the understanding of ‘crystal statistics’ in powder diffraction. The almost total dominance of the Bragg–Brentano geometry has resulted in the assumption that only large numbers of crystallites will give reliable intensities, which is only true if the scattering relied entirely on Bragg scattering events. There are also implications for the interpretation of orientation texture, since contributions from numerous reflections may be observed, despite not being in the Bragg condition. It suggests that the degree of texture could well be underestimated or biased because of the distribution of scattering in Ω and X. Based on this new theory, it has been possible to build and operate a small, high-resolution powder diffractometer, using a highly parallel beam of pure Cu *K*α_1_ and very small sample sizes and achieve reliable intensities (Fewster & Trout, 2013 ▶). It is hoped that this theory will help free-up the development of ideas in this area of research.

## Footnotes

1 The number of 5 µm radius crystallites irradiated during this experiment, by a beam of 35 µm wide and 256 × 55 µm pixels = 35 × 256 × 55/() × 40% = 2509.

## Appendix A

X-rays are scattered from the electron cloud, where there are levels of vibration that vary from the core to the shell. In strongly bonded Si the shell will be damped compared with the vibrations of the core, because it is linked *via* the bonding to other atoms (Reid & Pirie, 1980 ▶). The core Debye–Waller factor has been calculated and measured accurately, see for example the compilation and arguments in Reid & Pirie, and the measurements of Aldred & Hart (1973 ▶), who give a value close to the calculations at room temperature of *B* = 0.04613 nm^2^ using high-order reflections; this value is therefore more closely related to the core. This puts an upper limit on the value of *B* under these conditions. The calculated *B* associated with the shell is ∼0.021 nm^2^, slightly less than 0.5× for the core that is considered a good estimate. Reid & Pirie have considered the influence of directional bonding, making the *B* value a combination of an isotropic component ∼0.02455 nm^2^, and an anisotropic component arising from the distortions due to bonding. This anisotropic component is ∼−0.00631 nm^2^ factored with a reflection-dependent variable that is always less than unity. This again suggests that an approximated *B* value should be ∼0.02, and certainly not more than ∼0.046 nm^2^. The Debye–Waller factor has an exponential fall with increasing scattering angle, and since the measured and calculated intensities are scaled relative to each other up to the 444 reflection (the highest available for Si with the wavelength used in this study), a suitable value for *B* is required that captures the intensity fall-off over the whole experimental range. The shell model value of 0.02067 nm^2^ was used as a start, since this is also close to the dynamic deformation model of Reid & Pirie, although the agreement is little changed on using values up to 0.023 nm^2^, which is half the experimental value for the core biased value of Aldred & Hart.

This Debye–Waller factor is not the complete description, since the atomic vibrations are correlated due to the bonding. An understanding of this is important because this ‘thermal diffuse scattering’ (TDS) alters the distribution of intensity and has an impact on any description of the diffraction process that is concerned with modelling the profile. The detailed physics is given in James (1962 ▶); however, it suffices here to consider the vibrations as phonons of different wavelengths, ranging from the smallest separation between atoms up to the dimension of the crystallite. The dominant vibrations are longer wavelengths where groups of atoms move together, often termed acoustic phonons, whereas the high-frequency vibrations, *e.g.* adjacent atoms moving in opposite directions, are termed optical phonons. Each phonon creates a scattered wave that modulates the average, leading to satellites centred on the Bragg maximum. Since there are as many phonon wavelengths as there are repeat units in any one direction, these satellites merge to produce a redistribution of intensity from the Bragg peak that diminishes in magnitude further from the peak (decreasing in phonon wavelength). The fall in the TDS intensity from a Bragg peak follows a 1/*q* ^2^ form (Cochran, 1969 ▶) and is an incoherent fraction of the peak intensity (Harada & Sakata, 1974 ▶):

The variable α*_j_* is a complex function which includes the inverse of the square of the circular frequency of the *j*th mode at **q**, which can be related to the elastic parameters and is a function of **q**. However, this can be complicated and modified by data-collection strategies and temperature *etc*., for example, large detector aperture and effects of anisotropy (Stevens, 1974 ▶; Harada & Sakata, 1974 ▶); specimen size, experimental conditions and how the correction varies with sin θ/λ (Laktionov *et al.*, 1991 ▶); temperature changes and modification to the TDS/Bragg ratio (Reid, 1973 ▶). It is clear from this that the TDS contribution is a variable parameter that can be determined by experiment, since it modifies the intensity profile and background. The *I*_peak_ is the value that is modified by the Debye–Waller factor and the total intensity is unaltered, but just causes a redistribution of the intensity. These thermal vibrations are important to consider if just the peaks are measured and assigned to a structure factor.

Note 1   Experimental work on the GaN 0002 reflection from a 4 µm thick buffer layer on a (000*l*) sapphire substrate grown by metal organic chemical vapour deposition (MOCVD) is difficult to fit with dynamical theory in terms of height and width, but is much closer to kinematical theory, *i.e.* the peaks are narrower and higher than expected. GaN is known not to be perfect with threading dislocations along the growth direction, and although the growth process is very well controlled the quality from fitting to a substrate with a large mismatch lends itself to kinematical rather than dynamical theory. With the methods of sample preparation in powders with strains and distortions being unavoidable, it is likely that dynamical theory is inappropriate for typical powders.
